# Enhanced Photosynthesis and Carbon Metabolism Favor Arsenic Tolerance in *Artemisia annua*, a Medicinal Plant as Revealed by Homology-Based Proteomics

**DOI:** 10.1155/2014/163962

**Published:** 2014-04-29

**Authors:** Rashmi Rai, Sarita Pandey, Alok Kumar Shrivastava, Shashi Pandey Rai

**Affiliations:** ^1^Laboratory of Morphogenesis, Center of Advanced Study in Botany, Banaras Hindu University, Varanasi 221005, India; ^2^Laboratory of Algal Biology, Molecular Biology Section, Center of Advanced Study in Botany, Banaras Hindu University, Varanasi 221005, India

## Abstract

This paper provides the first proteomic evidence of arsenic (As) tolerance and interactive regulatory network between primary and secondary metabolism in the medicinal plant, *Artemisia annua*. While chlorophyll fluorescence and photosynthetic rate depicted mild inhibition, there was a significant enhancement in PSI activity, whole chain, ATP, and NADPH contents in 100 **μ**M As treatments compared to the control plants. However, a decrease in the above variables was recorded under 150 **μ**M treatments. Proteomic decoding of the survival strategy of *A. annua* under As stress using 2-DE followed by MALDI-MS/MS revealed a total of 46 differentially expressed protein spots. In contrast to other plants where As inhibits photosynthesis, *A. annua* showed appreciable photosynthetic CO_2_ assimilation and allocation of carbon resources at 100 **μ**M As concentration. While an increased accumulation of ATP synthase, ferredoxin-NADP(H) oxidoreductase, and FeS-rieske proteins supported the operation of cyclic electron transport, mdr ABC transporter protein and *pcs* gene might be involved in As detoxification. The most interesting observation was an increased accumulation of *LEAFY* like novel protein conceivably responsible for an early onset of flowering in *A. annua* under As stress. This study not only affirmed the role of energy metabolism proteins but also identified potential candidates responsible for As tolerance in plants.

## 1. Introduction

In recent years, metal/metalloid contamination has resulted in degradation of large areas of cultivable lands, posing a serious threat to sustainable agriculture and food security in developing countries like India [1]. The frequent use of As-laden ground water for irrigation elevates As levels in the soils and makes land unproductive and toxic, thereby resulting in severe reduction of growth and yield of food crops [2, 3]. This calls for judicious management of contaminated lands to put them back in cultivation. Although a number of As-tolerant noncommercial plants, including some species of the genera* Pteris, Agrostis, Lepidium, Lemna, *and* Jasione,* are known to accumulate high levels of As and have been successfully exploited for As phytoremediation [4, 5], none of these have any commercial use. Hence, it is highly desirable to identify nonedible plant species which may not only be cultivated for phytoremediation of As contaminated soils but also put back into commercial use.

*Artemisia annua *(Asteraceae) is an important dicotyledonous medicinal plant native from China and widely distributed in temperate and subtropical zones of the world, especially in Asia. It is a nonmodel plant with limited genomic information, although sequencing of limited number of randomly selected cDNA clones has been achieved [6]. Despite the above, this plant has provoked wide interest for biotechnological studies because it produces an antimalarial drug, called artemisinin. Currently, artemisinin and its semisynthetic derivatives are extensively used in the treatment of malaria, mostly in combination therapies [7], as well as in the treatment of cancers and viral diseases [8, 9]. In addition to the therapeutic properties,* A. annua *possess important attributes such as a fast growth rate and a high biomass production, easy cultivation, and an unusual weedy habit, all of them important traits for plants employed for phytoextraction of metals and metalloids from contaminated soils [10]. Our previous study demonstrated a significant induction of artemisinin production under As stress. The high tolerance of* A. annua *against As stress was coupled with an appreciable increase in biomass and As accumulation capacity, thus enabling this plant to be worth cultivating in As contaminated soils [11].

The enhanced artemisinin production and As tolerance of* A. annua* raises the question as which proteins could be potentially responsible for the above traits and how far the changes in different metabolic pathways, that is, primary and secondary, are interrelated with each other. While some mechanisms including, surface exclusion, intracellular detoxification, efflux, and production of phytochelatins have been proposed for the development of As tolerance in plants [12], the molecular mechanism underlying physiological changes still remains to be elucidated. Since proteins are directly involved in stress responses, any change in biochemical and physiological parameters is likely to be reflected at the proteome level. Proteomics has become a useful tool to unravel the relationships between protein abundance and stress acclimation [13]. High resolution 2D-PAGE (2-DE) is the most direct approach for defining gene functions, and studies based on transcriptomics and proteomics can provide significant information on the processes involved in these stress responses [14].

Proteomics has been widely used to investigate the proteome of plants under abiotic stresses [15–17]. However, proteomic studies on As-induced changes are still few and limited to maize roots [18], maize shoots [19], rice roots [20], rice leaves [21], a pseudometallophyte* Agrostis tenuis* [22], and the hyperaccumulator species* Pteris vittata* fronds [23] and roots [24]. With the exceptions of* Agrostis *and* Pteris*, all the above mentioned proteomic investigations primarily focus on plants which are agronomically important food crops. It is worth mentioning that country like India, where food-security is of foremost concern, to use such plants for phytoremediation of As-contaminated agricultural lands cannot be acceptable. In view of the fact that none of above plant species is well adapted to high As concentrations,* A. annua *emerged as the plant of choice. Despite potential application and tolerance the proteome of A. annua has remained unexplored due to unsequenced genome and inadequate representation in annotated, nonredundant (nr) protein databases. This study is the first attempt to decode the As tolerance strategy of* A. annua *combining physiological and proteomic analyses and to establish interactive network between its primary and secondary metabolism.

It requires mentioning that As (V) exposure (i) triggers ROS production causing alterations in the antioxidant pathways [11, 20], (ii) disturbs phosphate metabolism (e.g., ATP synthesis) and electron transport systems [12], and (iii) induces photoinhibitory conditions [25, 26]. In view of the above it was hypothesized that for survival under As stress,* A. annua* may (i) increase the accumulation of APX and DHAR to detoxify ROS, (ii) increase the electron transport to overcome the shortage of ATP and NADPH, and (iii) enhance phytochelatin synthase (*pcs*) and ABC transporter like protein genes for detoxification.

Taking recourse to the above the present study was undertaken to have an insight into (i) the metabolic proteins involved in As tolerance and to ascertain how far the physiological attributes go hand in hand with proteomic data and (ii) establish protein network between primary and secondary metabolism pathways. The results of this study enabled us to associate physiologically significant candidate proteins with As stress tolerance mechanism in* A. annua*.

## 2. Materials and Methods

### 2.1. Hydroponic Dose Screening

*Artemisia annua *L (family: Asteraceae) plants were cultured hydroponically as described by Rai et al. [11]. Briefly, seeds of* A. annua* were collected from Central Institute of Medicinal and Aromatic Plants (CIMAP), Lucknow, India, and grown in botanical garden, Banaras Hindu University, Varanasi, India. Twenty one days after germination, seedlings of equal size (approximately 10 cm) were washed with deionized water and acclimated in a hydroponic system in 10% Hoagland's nutrient solution [27]. The nutrient solution was aerated continuously using an aquarium pump and renewed twice a week. Seedlings were kept in a controlled room with a 14 h light period, 100 *μ*mol m^−2^ s^−1^ photosynthetic photon flux density (PPF), 28/26°C day/night temperature, and 60–70% relative humidity. After one week, the plants were transferred into 10% strength Hoagland's nutrient solution, spiked with different doses such as 50, 100, and 150 *μ*M of As. Arsenate was applied as Na_2_HAsO_4_·7H_2_O. Experiments were conducted in triplicate in 150-mL Erlenmeyer flasks containing 100 mL of nutrient solution. Each replicate contained 3 plants of equal height (approximately 10 cm). Control seedlings were never treated with As. Three replicates, each including the two concentrations and the control, were placed separately in growth chamber and were harvested after 7 days of treatment. After 7 d of As exposure no visible toxicity symptoms were seen up to 100 *μ*M. Moreover, lower concentrations of As stimulated root growth and the biomass the maximum being 21.3 and 44.4%, respectively, at 100 *μ*M As over the control. However, toxicity symptoms appeared and the root length decreased by (6%) at 150 *μ*M. This clearly indicated that plants fail to tolerate As beyond 100 *μ*M (data not shown). Based on these data, the diagnostic concentrations for the proteome analysis were set at 100 and 150 *μ*M. Moreover, these doses represent the likely range of arsenic in contaminated lands of eastern Uttar Pradesh (U.P), India [28].

### 2.2. Pot Culture Experiment

Based on the results of the hydroponic study another set of pot culture experiments were conducted in natural conditions (field) using soil treated with similar doses of As, 100 and 150 *μ*M As in the form of sodium arsenate. Experiments were done in triplicate. The soil used for the pot experiments was alluvial, collected from the premises of botanical garden, Banaras Hindu University, Varanasi, India. The soil was mixed with sand at the ratio of 3 : 1 (v/v). As solution was uniformly mixed with air-dried soil, kept for one month to stabilize and filled in 16 kg pots. The soil without As treatment was used as control. Four seedlings of twenty-one day-old healthy* A. annua *were transplanted into each experimental pot. Soil moisture was maintained at 50–60% of field water holding capacity by adding water during the experimental period. Plants were grown under natural daylight and ambient temperature for eight months, after which they were harvested for protein isolation as well as to investigate the effects of As on flowering time, inflorescence, and other morphological as well as physiological characters.

### 2.3. Extraction and Estimation of Arsenic and Phosphate

Total As was estimated in an atomic absorption spectrometry (Perkin-Elmer; A Analyst 600) fitted with a graphite furnace [29]. The As reference standard of 1000 mg mL^−1^ (AA03N-5) was supplied by Accustandard, USA. Total phosphate was extracted from shoots of control and As treated seedlings according to the method of Fiske and Subbarow [30]. Absorbance was measured at 660 nm using KH_2_PO_4_ as a phosphate standard.

### 2.4. Measurements of Photosynthesis and Chlorophyll Fluorescence

Photosynthetic rates (*P*_*s*_), stomatal conductance (*g*_*s*_), intercellular CO_2_ (*C*_*i*_), water use efficiency (WUE), and transpiration rates (*E*) of test plants were determined using a LI-6400, (LICOR Inc., Neb., USA) portable gas exchange system on the uppermost fully expanded leaves from each individual during the period from 8:30 a.m. to 10:30 a.m. in six randomly selected plants of each treatment. During the experiments, system was calibrated with known CO_2_ concentration of 509 ppm and PAR ranged between 1000 and 1200 *μ*mol m^−2^ s^−1^; Chlorophyll fluorescence was determined with a portable plant efficiency analyzer (Model PEAMK2 9414, Hansatech Instrument Ltd., Norfolk, UK) on the same leaf where (*P*_*s*_) was measured. WUE was calculated as the ratio of *P*_*s*_ and *E*. Leaf clips for dark adaptation were placed on the adaxial side for 15 min before measurement at excitation irradiance of 2000 *μ*mol m^−2^ s^−1^. Minimum fluorescence (*F*_*o*_) and maximum fluorescence (*F*_*m*_) were measured. Variable fluorescence was calculated as (*F*_*v*_ = *F*_*m*_ − *F*_*o*_) and the ratio of variable to maximum fluorescence as (*F*_*v*_/*F*_*m*_).

### 2.5. Measurement of ATP, NADP, PSI, PSII, and Whole Chain Activity

ATP and NADPH contents as well as PSI and PSII activities were measured on the uppermost fully expanded leaves of each treatment and control (sampled in six). The ATP pool was measured by the method of Larsson and Olssonl [31]. ATP was quantified using Luminometer (LKB, 1250). The NADPH/NADH level of the cell extract in Tris-Cl (pH 8.0) was measured by recording the absorbance at 340 nm [32]. PSI- and PSII-mediated electron transport were measured using isolated thylakoids with an oxygen electrode (digital oxygen system model-10, Rank Brothers, UK). Thylakoid was isolated from leaves using a modified protocol of Mishra and Sabat [33]. Leaves were crushed in extraction buffer containing 300 mM sucrose, 10 mM NaCl, 10 mM CaCl_2_, 5 mM MgCl_2_, 10 mM ascorbate, and 20 mM Tricine-NaOH (pH 7.5). The homogenate was filtered and centrifuged at 6000 g for 10 min. The pellet represents the thylakoid fraction and was suspended in 1 mL of buffer containing 100 mM sucrose, 10 mM NaCl, 10 mM CaCl_2_, 5 mM MgCl_2_, and 20 mM Tricine-NaOH (pH 7.5). The reaction mixture for assaying PSII activity consisted of 50 mM HEPES buffer (pH 7.5) containing 400 mM sucrose, 5 mM MgCl_2_, and 10 mM NaCl in a final volume of 1 mL. The activity was measured as oxygen evolution with water as electron donor and 500 *μ*M phenyl* p*-benzoquinone (pBQ) as the electron acceptor [34]. The whole-chain (H_2_O–MV) electron transport was measured polarographically as O_2_ uptake. The 1 mL reaction mixture contained 20 mM HEPES (pH 7.5), 100 mM sucrose, 10 mM NaCl, 2 mM MgCl_2_, 0.5 mM MV, and 1 mM sodium azide. In the third type of assay, the electron transport through PSI was also measured polarographically as above except that reduced DCPIP was used as the electron donor. Assay conditions were identical to those of whole chain assay except that 5 *μ*M DCMU (3-3,4-dichlorophenyl-1,1-dimethylurea), 1 mM sodium ascorbate, and 100 *μ*M DCPIP were added to the above reaction mixture. A thylakoid aliquot containing 10 *μ*g chlorophyll was used in the measurements. The chlorophyll content was estimated according to Arnon [35]. All the photochemical assays were carried out at a saturating intensity of white light (1000 *μ*E m^−2^ s^−1^) at 26 ± 2°C.

### 2.6. Determination of Artemisinin and Anthocyanin Contents

Artemisinin was extracted and quantified by the method of Zhao and Zeng [36]. Concentration of artemisinin was measured using calibration curve of artemisinin standard. Anthocyanins were extracted from 1 g of fresh leaves with 20 mL of mixture: n-propanol : HCl : H_2_O (18 : 1 : 81, v/v/v) according to Lange et al. [37] and Bette and Kutschera [38] in modification. Samples were heated in a boiling water bath for 10 min and then incubated for 24 h in the dark at 4°C. The extract was filtered and its absorbance was measured at 529 and 650 nm. The red peak absorbance for degraded chlorophyll in the sample extracts occurs at 650 nm and has a tail that overlaps with the anthocyanin peak at 529 nm. Therefore, the absorbance of anthocyanin was corrected for the effect of chlorophyll using this equation: AA =* A*_529_–(0.288* A*_650_) where AA is corrected anthocyanin absorbance. Total anthocyanin content was then calculated using this corrected absorbance and a molar absorbance coefficient for anthocyanin at 529 nm of 30,000 mol^−1^ cm^−1^ The content of total anthocyanins was expressed as *μ*mol per g FW [39].

### 2.7. Protein Extraction and 2-DE Separation

The experiments were conducted in triplicate. Three plants per treatment (both 100 & 150 *μ*M) and control were collected after 8 months of pot culture experiment. Two leaves per replicate (2nd pair and weighing 0.5 to 1.0 g fresh weight) were ground in liquid nitrogen to a fine powder. The powder was suspended completely in 10 mL of TCA extraction buffer (10% w/v TCA in acetone with 0.5% w/v *β*-mercaptoethanol) at −20°C overnight. After centrifugation at 6000 ×g, for 30 min at 4°C the pellets were washed 3 to 4 times with 10 mL ice cold acetone (with 0.5% w/v *β*-mercaptoethanol). The collected protein pellets were dried in air. The dried powder was resuspended completely in 2 mL lysis buffer containing 7 M urea, 2 M thiourea, 4% CHAPS, 40 mM DTT, and 1.0% IPG buffer (4–7). Traces of bromophenol blue were added and the solution was centrifuged at 19000 ×g for 10 min. Protein contents were quantified according to Bradford using BSA (bovine serum albumin) as a standard (2 mg mL^−1^) [40]. Sample entry was made through in-gel rehydration. A total of 300 *μ*L of solubilization buffer containing 250 *μ*g protein sample was incubated with the dry IPG strips (pH 4–7; 13 cm; GE healthcare, USA) at 20°C for 16 h. The first dimension separation was conducted at 20°C with an Ettan IPGphor system (GE Healthcare BIO-Science, USA). Focusing was performed in 7 steps: linear 30 V for 00:30 h, 150 V for 2:00 h, 300 V for 00:40 h, 500 V for 4 h, gradient 1000 V for 1 h, gradient 8000 V for 2 h, and finally 8000 V for 13:00 h. Focused IPG strips were then equilibrated by first incubating them in an equilibration solution (6 M urea, 30% v/v glycerol, 2% w/v SDS, 50 mM Tris-HCl, pH 8.8, and 1% w/v DTT and a trace amount of bromophenol blue) for 15 min, followed by incubation in 2.5% w/v iodoacetamide in the same equilibration solution instead of 1% DTT for 15 min. The strip was placed on the top of 12.5% SDS-PAGE (13 × 15 cm) and sealed with 0.5% agarose. Electrophoresis was carried out at 10 mA/gel for 30 min then 25 mA/gel for 5 h using a Hoefer SE 600 apparatus (Amersham Biosciences, USA). The gels were stained with CBB R-250 as described by Pandey et al. [41]. Three replicates of CBB stained gels from three independent biological samples were used for analysis.

### 2.8. 2-DE Gel Data Analysis

Gels were scanned and protein spots were analyzed by using PDQuest software version 7.1 (Bio-Rad). After background subtraction and spot detection, spots were matched. Missing values imputation was done by K-Nearest Neighbor (KNN) algorithm [MATLAB]. After missing values imputation, total spot intensity per gel was used to normalize spot intensities (% of individual spot intensity/Σ% spot intensity of each gel) to compensate for variations between gel replicates. These data sets were also log-transformed to reduce the spot volume-spot deviation dependency. Only spots with statistically significant (Duncan Multiple Range Test, DMRT, *P* < 0.05) and reproducible changes were considered valid, and the protein spots with an abundance ratio of at least 1.5-fold were selected as differentially expressed proteins.

### 2.9. In-Gel Digestion and Peptide Extraction

46 differentially expressed protein pots from CBB stained gels were manually excised and in-gel digestion was performed as per the protocol of Bruker Daltonics adapted from Schevchenko et al. [42]. 100 mL of washing solution (1 : 1 ratio of acetonitrile and 100 mM ammonium bicarbonate) was added to excised spots and incubated for 30 min for destaining. It was further incubated in absolute acetonitrile for 3 min and pellets were air-dried. Reduction of the protein spots was done using 10 mM DTT dissolved in 100 mM NH_4_CO_3_ for 1 h at 50°C. The samples were further alkylated using 55 mM iodoacetamide in 100 mM NH_4_CO_3_ for 45 min in dark at room temperature. The gel pieces were dehydrated with 100% acetonitrile, air-dried, and then digestion was done by using sequence-grade modified trypsin (Promega gold) which was added (25 *μ*g/mL) in a digestion buffer containing 25 mM ammonium bicarbonate to the dehydrated gel pieces. In-gel digestion of protein was performed overnight at 37°C, followed by three extractions of peptides with 50% (v/v), acetonitrile 0.1% (v/v), and trifluoroacetic acid (TFA). Combined supernatants were dried in a vacuum centrifuge (Thermo electron corporation, UVS400A) for 2–4 h. Dry pellets were resuspended in a small volume (3–5 mL) of 0.1% (v/v) TFA in 50% (v/v) acetonitrile and stored at −20°C until analysis.

### 2.10. Mass Spectrometry Analysis and Database Searching

For MS analysis, one microliter of alpha cyanocinnamic acid (CCA) matrix (10 mg/mL in 50% acetonitrile and 0.1% TFA) (Sigma Aldrich, St. Louis, Missouri, USA) was mixed with 1 *μ*L of digested protein samples. The mixture was spotted on MALDI target plate (MTP 384 ground steel, Bruker Daltonics, Germany) and allowed to form the crystals. The peptide spectra was acquired in an AUTOFLEX speed MALDI TOF/TOF instrument (Bruker Daltonics, Germany) having Nd: YAG smart Laser beam of 335 nm wavelength. External calibration was done with peptide calibration standard supplied by Bruker, with masses ranging from 1046 to 3147 Da. The obtained spectra were acquired using FlexControl version 3.3 software (Bruker Daltonics, Germany) in reflectron ion mode with an average of 2000 laser shots at the m/z detection range between 700 and 4000. The peak list was generated automatically and at least their most intense and discriminating peaks were subjected to further MS/MS fragmentation in LIFT mode.

Accelerating voltage:

 Ion Source-1 19 kV and Reflectron (grid)-1 21 kV
 Ion Source-2 16 kV and Reflectron (grid)-2 9.65 kV.

The data were analyzed using Flex Analysis software version 3.3 (Bruker Daltonics, Germany) and searched in MASCOT web server (Matrix Science; http://www.matrixscience.com/) using Biotools version 3.2 software (Bruker Daltonics, Germany). The protein databases employed were NCBInr (The National Center for Biotechnology Information nonredundant) and Swiss-Prot (date of access, 5. 11. 2012). A combined peptide mass fingerprinting (PMF) and tandem MS/MS were performed with the following MASCOT settings: taxonomy as* Viridiplantae*; peptide mass tolerance of ±100 ppm for peptide mass fingerprinting and ±1.2 Da for MS/MS, monoisotopic mass, alkylation of cysteine by carbamidomethylation as a fixed modification and oxidation of methionine as a variable modification. Proteins with threshold MASCOT score >43 (*P* < 0.05) were considered as significant match. The peptide sequences with highest scores of each spot were validated by BLAST P analysis. Also, the borderline MASCOT hits that showed poor score and those annotated as unknown, predicted, or hypothetical proteins were further searched in BLAST P analysis against the NCBI nr database to query their homology.

### 2.11. RNA Isolation and DNase Treatment

Total RNA was isolated from the control and As treated leaf samples in TRIZOL reagent (GIBCO-BRL) using the instructions given in the manufacturer's protocol. Briefly, 100 mg of leaf samples was ground in liquid nitrogen and mixed with TRIzol reagent. After centrifugation at 12,000 g for 15 min at 4°C, the upper aqueous layer containing RNA was removed and placed in a new centrifuge tube. About 0.5 mL of isopropyl alcohol per 1 mL of TRIzol was used to precipitate RNA. This suspension was centrifuged at 12,000 g for 8 min at 4°C and the pellet washed with 75% ethanol and resuspended in 50 *μ*L DEPC treated water. Forty units of RNase free DNase was added to remove any contaminating DNA followed by heating at 70°C for 5 min to denature the DNase. The quality of RNA was checked on a 1.0% agarose gel and the concentration determined by measuring the absorbance at 260 nm.

### 2.12. Primer Design and RT-PCR Analysis

The cDNAs of DXS, DXR, HMGR, GPPS, FPS, and CHS were synthesized using RT-PCR based on the* A. annua* nucleotide sequences published in database. The database employed was GenBank (GenBank accession numbers AF182286, AF182287, AF142473, and AF112881, AF112881, resp.). The sequences of the primers used for PCR amplifications are listed in Supplementary Table S2 in Supplementary Material available online at http://dx.doi.org/10.1155/2014/163962. Amplification was performed with initial denaturation at 94°C for 5 min, followed by 30 cycles of 30 s at 94°C, 30 s at 54°C, 90 s at 72°C, and a final extension at 72°C for 10 min. Ribosomal protein S9 (RPS9) was used as an internal reference gene. The amplification of each gene was repeated at least twice. Since the genome of* A. annua* is largely unsequenced, to design the gene specific primers, such as of GAPDH and PAL, the highly conserved region was picked up through multiple sequence alignment of different dicot sequences available in database by using Primer 3 software and these primers were used to amplify genomic DNA from* A. annua*. Specificity of all the primers was confirmed by sequence analysis of RT-PCR amplicons derived from* A. annua.*

### 2.13. Western Blot Analysis

SDS-PAGE was carried out as per the method of Mishra et al. [43]. 20 *μ*g protein was separated by SDS-PAGE and electroblotted onto PVDF membrane (Millipore Immobilon-P), using a dual Mini-electroblot system (Precision Instruments, Varanasi, India) as described [44]. The gel cassette was kept in transfer buffer 3.03 g/L Tris base, 14.4 g/L glycine, and 200 mL/L methanol (99% v/v pure) for 12 h or overnight at 15 V at 4°C. Membrane was blocked for 4 h in TTBS (Tris buffer saline containing 0.1% Tween-20) and 5% (w/v) nonfat dried milk. The primary antibody (anti-PCS antibody) was diluted as per the instructions of the donors. The membrane incubated overnight at 4°C in the diluted solution of the primary antibody was washed five times for 5 min each in TTBS. This was then incubated in a Goat anti-Rabbit IgG HRP (horseradish peroxidases) conjugated secondary antibody (Genei, India) for 4 h. Following four consecutive 5 min wash in TTBS the membrane was developed with DAB/NiCl_2_ visualization solution. The reaction was terminated by washing the PVDF membrane with deionized distilled water. The blots were dried between filter paper, which considerably reduced the background staining. Polyclonal antibodies used for the detection of PCS by immunoblotting were obtained as generous gift from Dr. Stephan Cuine (CEA Cadarache, France). The immunoblotswere photographed using a gel documentation system (Bio-Rad, USA) and their quantitative densitometric analysis was performed using the Quantity One Software (Bio-Rad, USA).

### 2.14. Statistical Analysis

The statistical significance of the data was analyzed using one-way ANOVA, followed by Duncan's new multiple range test (DMRT) using the statistical software package SPSS 12.0. The results were accepted as significant at *P* < 0.05. All values shown in the figures and tables are means ± SD. For statistical treatment and cluster analysis of protein abundance values, the web-based software NIA array analysis tool used was Sharov et al. [44] available at http://lgsun.grc.nia.nih.gov/anova/index.html. This software tool selects statistically valid protein spots based on analysis of variance (ANOVA). The data (MS excel sheet with indication of biological replications) were statistically analyzed using the following settings: error model “max (average, actual),” 0.01 proportion of highest variance values to be removed before variance averaging, 10 degrees of freedom for the Bayesian error model, 0.05 FDR threshold, and zero permutations. Hierarchical clustering was performed to check the reproducibility of data set (based on 39 differentially expressed protein spots), and the results were represented in dendrograms using the Euclidean distance. PCA was done using the following settings: covariance matrix type, three principal components, 1-fold change threshold for clusters, and 0.6 correlation threshold for clusters. PCA results were represented as a biplot, with proteins with similarity in expression pattern located in the same area of the graph.

## 3. Results 

### 3.1. Arsenic and Phosphate Concentrations

*A. annua *accumulated As in a concentration dependent manner in both root and shoots. Maximum As content was found to be (191.01 *μ*g g^−1^ DW) in 150 *μ*M As treatment as compared to the control (6.08 *μ*g g^−1^ DW). In contrast, phosphate concentration showed a dose dependent decrease with lowest value in the 150 *μ*M As treated plants as compared to the control (Supplementary Table S1).

### 3.2. Morphological Responses of* A. annua* to As Stress

**Figure 1:**
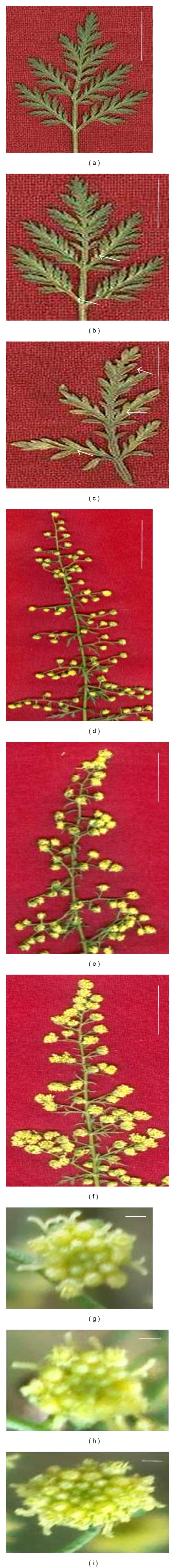
Morphological and physiological alterations in* Artemisia annua* seedlings; anthocyanin deposition in leaflets as indicated by white arrows; control (a), 100 *μ*M (b), 150 *μ*M As treatments. Number of inflorescence and inflorescence branches; control (d), 100 *μ*M (e), and 150 *μ*M As treatments (f). Florets size and number; control (g), 100 *μ*M (h) under 150 *μ*M As treatment (i). A B C bar indicates 4 cm, D E F bar indicates 2.5 cm, and G H I bar indicates 1 mm.

Upon visual inspection,* A. annua* plants did not show any phytotoxicity symptoms at 100 *μ*M As concentration. The plants looked healthy and green with an increased biomass, affirming their tolerant nature (Figure 1, Table 1). In contrast, 150 *μ*M As treated plants showed visible toxicity symptoms such as wilting, whitish chlorosis in young leaves and yellowing and necrosis in old leaves (Figure 1(c)). The most emblematic symptoms observed under 150 *μ*M As treatments were intense red coloration of leaflets particularly at the apex (Figure 1(c)). The pigment analyses from leaves of comparable developmental stages revealed a 3.0-fold higher anthocyanin content in 150 *μ*M As treated plants compared to the control (Table 1). The data compiled in Table 1 depicted an inhibitory effect of As on plant height, number of inflorescence, and inflorescence branches being more pronounced at 150 *μ*M As concentrations. Interestingly, however, the number of florets, the size of capitula, the ratio of capitula/inflorescence, and the number of oil glands were found to be increased significantly under As treatments conceivably contributing to the higher artemisinin and essential oils production in As treated plants (Table 1, Figures 1(h) and 1(i)). Another remarkable observation made was the early onset of flowering in As treated plants. As treated plants flowered about 4-5 weeks before than control plants after sowing (Table 1).

### 3.3. Physiological Responses of* A. annua* to As Stress

Table 2 compiles data on the various physiological traits such as photosynthetic rate (*P*_*s*_), stomatal conductance (*g*_*s*_), transpiration rate (*E*), intercellular CO_2_ concentration (*C*_*i*_), water use efficiency (WUE), and chlorophyll fluorescence emission patterns (*F*_*v*_/*F*_*m*_) of control and As treated* A. annua *plants. While in the 100 *μ*M As treatments *P*_*s*_ was found to be slightly depressed by (4.7%) and was near the control, the 150 *μ*M As treatments resulted in a reduction of the leaf photosynthetic activity by 26% with respect to the control. With regard to *g*_*s*_, the value of this parameter decreased by 18% and 23% in 100 and 150 *μ*M As treatments, respectively. However, in contrast, the intercellular CO_2_ concentration showed a little increase under 100 *μ*MAs treatments, although was equivalent to the control Transpiration rate demonstrated a negligible decrease under As treatments. An increase in the water use efficiency (WUE) due to As stress was observed in both treatments, being 37% and 34% of the control in 100 *μ*M and 100 *μ*M As treatments, respectively. Considering photosynthetic electron transport chain activities, PSII demonstrated a minor decline of 11% and 34% in 100 *μ*M and 150 *μ*M As treatments, respectively, whereas PSI showed a significant increase of 70% in 100 *μ*M As treatments. In addition, the chlorophyll fluorescence parameters were monitored to determine the performance of photosystem 2 (PSII). The *F*_*v*_/*F*_*m*_ ratio is correlated with the efficiency of leaf photosynthesis and a decline in this ratio is a good indicator of photoinhibitory damage under stress [45]. The *F*_*v*_/*F*_*m*_ ratios in the 100 *μ*M As treatments were slightly below 0.83, which is the theoretical optimum [46]. In contrast it dropped down to 0.72 in the plants exposed to 150 *μ*M of As, indicating some damage in the PSII [47].

### 3.4. Biochemical Responses of* A. annua* to As Stress

Biochemical parameters including anthocyanin, artemisinin, ATP, and NADPH contents were measured from 8-month-old plants (Tables 1 and 2). Anthocyanin content was increased by 175% and 275% in 100 and 150 *μ*M As treatment, respectively. In tune with the above, the concentration of artemisinin was increased by 53.1 and 75% in 100 and 150 *μ*M As treatments, respectively, over the control (Table 1). While in 150 *μ*M As treatments, both NADPH and ATP contents decreased by 24% and 29%, NADPH registered an increase of 29% in 100 *μ*M As treatments, but insignificant increase in ATP content was observed (Table 2).

### 3.5. 2-DE Analysis of As-Responsive Leaf Proteins in* A. annua*

**Figure 2:**
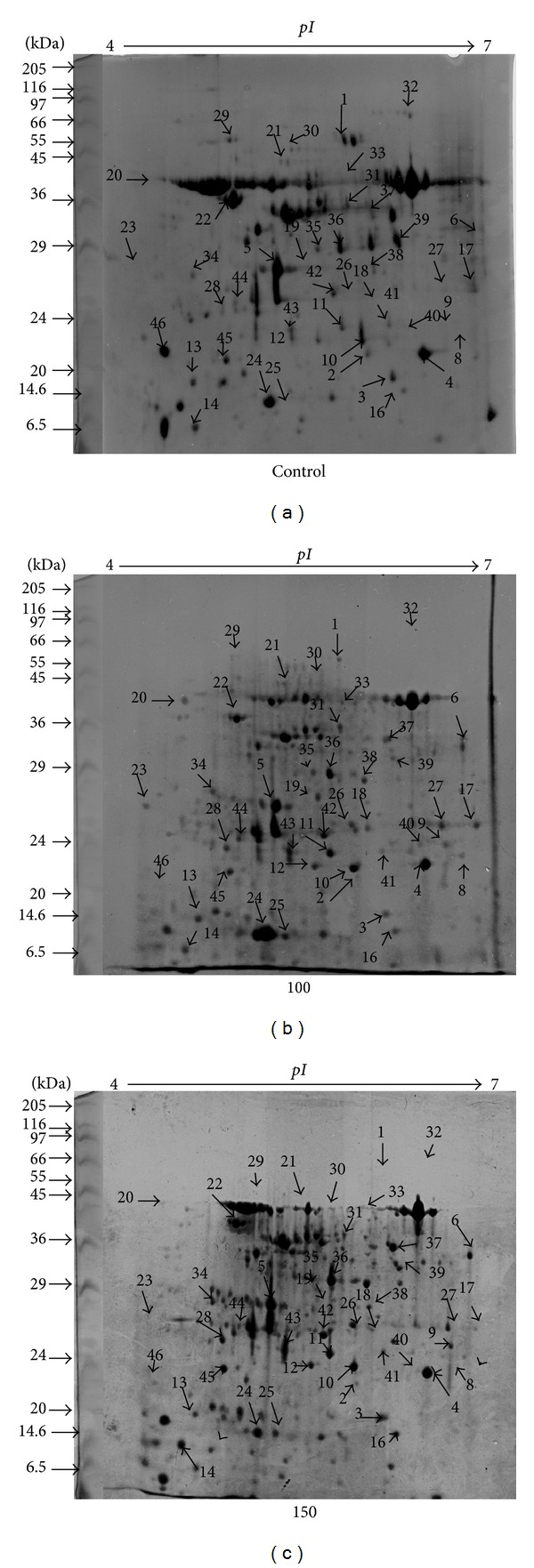
The 2-DE images of cytosolic protein extracts from* Artemisia annua* (a) control, (b) 100 *μ*M, and (c) 150 *μ*M As. All gels were run in triplicate. Proteins were extracted and separated by 2-DE and visualized by CBB staining. The protein (250 *μ*g) was applied to pH 4–7 IPG dry strips with 12.5% linear vertical SDS-PAGE as the second dimension. The arrows with numbers on 2-DE gel indicate the differentially expressed proteins, which were further identified by MALDI-MS/MS analysis. The molecular mass marker (Sigma) and* pI* are indicated on the left side and above the gels, respectively.

Figure 2 presents a comparative account of the CBB stained gels of untreated control and 100 and 150 *μ*M As treated* A. annua* seedlings after 8 months of treatment. Quantitative image analysis using PD Quest Version 7.1 software (Bio-Rad) from three biologically independent replicate experiments revealed a total of 391 ± 35, 412 ± 41, and 447 ± 27 spots in the control, 100 *μ*M and 150 *μ*M As treatments in the range of pH 4–7, and relative molecular masses 14–90 kDa. Supplementary Table S3 contains the description of the 2-DE proteomic experiment.

Fifty-one protein spots consistently changed their abundance (vol. %) by ≥1.5-fold in at least one of the As treatments. However, a spot displaying control versus treatment ratio of at least 1.5-fold and consistently present or absent in all three replicates was considered valid. Fold change was calculated as a ratio of the averaged means of normalized spot volumes of control and the treatments. Essentially arbitrarily threshold values ranging from 1.3 to 2.0 fold have been used in earlier proteomic studies [48, 49]. Nevertheless, a 1.5 threshold value was selected in order to focus on the most responsive proteins and for consistency with previous proteomic and microarray experiments [18, 41, 50]. While most spots showed quantitative changes, few protein spots demonstrated qualitative changes. For example, spots 29, 30, and 32 were absent in As treated gels at one or more time points, whereas spots 9, 14, 39, and 40 were absent in the control gels (Supplementary Table S3). Raw data was processed in three steps, missing value estimation, normalization, and transformation. Analysis of variance before and after data transformation is given as Supplementary Table S4.

### 3.6. MALDI-TOF/TOF Analysis, Functional Classification, and Identification of As-Responsive Proteins

**Figure 3:**
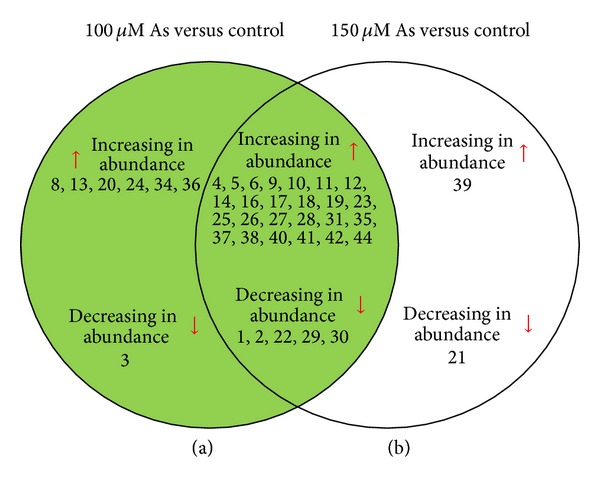
Venn diagram representation of differentially expressed protein spots of* Artemisia annua *affected under different As treatments. (a) and (b) represent 100 and 150 *μ*M As treatments versus control, respectively. Increase and decrease in abundance of protein abundance are indicated by the upward and downward arrows, respectively. Protein spots in the overlap region were common in both 100 and 150 *μ*M As treatments (a *∩* b).

Forty-six differentially expressed protein spots (based on *P* ≤ 0.05) with reproducible alterations were subjected to MALDI-TOF/TOF analysis (Supplementary Table S5).  Figure 3 shows the Venn diagram of the differentially expressed proteins at different concentrations of As. From 46 spots excised, 37 were successfully identified; two were not hit (spots 7 and 15) whereas 7 proteins were annotated either as unknown, putative, or hypothetical proteins (Table 3, Supplementary Figure S1). To gain functional information about these proteins, BLASTP (http://www.ncbi.nlm.nih.gov/BLAST) was used to investigate their homologies with other proteins in the database. Of the seven proteins, three were successfully BLAST identified (spots 5, 20, and 23); however, spots 32, 33, 43, 45, and 46 showed a poor BLAST score. Since the genome of* A. annua* is still unsequenced therefore the MS/MS results (the peptide sequences with highest scores) of each spot were also validated by BLAST P analysis which revealed similarity thereby validating the MS/MS results. These proteins were sorted into 7 functional groups including energy metabolism, ROS scavenging and defense, secondary metabolism, transcriptional regulator, protein metabolism, transport, and unknown/unidentified proteins according to the ontological classification of Bevan et al. [51] (Supplementary Figure S2). BLAST search results are given as Supplementary Table S6.

### 3.7. Expression Profiles of Proteins Associated in Different Metabolic Pathways

Expression pattern of selected proteins of C, control, and 100, 150 *μ*M As treatmentsis depicted in Supplementary Figure S3. Few proteins were represented by more than one spot with slightly different Mr and pI values, although they were excised from the same gel. For example, Chl a/b type I was identified in four spots (2, 10, 11, and 20), NADP-ferredoxin oxidoreductase was identified twice (8 and 38), carbonic anhydrase was identified in three spots (17, 27, and 40), triose-phosphate-isomerase (spots 42 and 44), and glyceraldehyde-3-phosphate (spots 6, 23, and 37). Most of the protein matches and theoretical and experimental pI and Mr were in good agreement, supporting confidence in the identifications; however, some of the identified proteins showed discrepancy with their Mr and pI. These kinds of phenomena are commonly observed in 2-D gels for several reasons including protein migration, different isoforms derived from different genes, proteolytic cleavage, posttranslational modification variants of the same gene product, or artificial modification of proteins, such as carbamylation during protein extraction [52]. According to the expression pattern, the identified proteins are categorized as proteins showing increased or decreased abundance under 100 or 150 *μ*M As treatments. More than any functional group, energy metabolism proteins (60%) were differentially expressed in 100 *μ*M As treatment and were divided into four functional subgroups: (1) chlorophyll a, b-binding proteins, (2) oxygen-evolving complex proteins of PSII, (3) proteins participating in the Calvin cycle, and (4) proteins involved in photosynthetic electron transport chain. The first subgroup harbored four protein species of chlorophyll a/b binding proteins (CP26 in PS II) displaying a mixed pattern of expression. While two isoforms of chlorophyll a/b binding protein (spot 10 and spot 20), an unknown protein that was BLAST identified as chlorophyll a/b binding protein, were found in higher amounts in either 100 or 150 *μ*M As treatments, spot (2) was decreased in relative abundance in response to As stress. Another protein (spot 11) identified as chloroplast PS I type III chlorophyll a/b binding protein was increased in relative abundance in 100 *μ*M As treatment. The second subgroup consists of oxygen-evolving complex protein (OECP2) where one intact protein (spot 4) was increased in relative abundance (2.3-fold in 100 *μ*M and 2.2-fold in 150 *μ*M), whereas one degraded unnamed protein product (spot 5) BLAST identified as oxygen-evolving complex protein (OEC1) was decreased in amounts (1.3-fold and 1.2-fold in 100 and 150 *μ*M As, resp.). The third subgroup consisted of 15 proteins. Most of the enzymes involved in different metabolic pathways such as plastidic aldolases (ALD, spot 12, 2.69-fold, and spot 36, 1.5-fold), fructose bisphosphatase aldolase class I (FBA, spot 36, 1.5-fold), glyceraldehyde 3-phosphate dehydrogenase (GAPDH, spot 6, 1.8 fold, spot 23, 3.1 fold, and spot 37, 1.6 fold), Ribose-5-phosphate,3-epimerase (spot 41, 1.7 fold), triose-phosphate-isomerase (spot 42, 1.6 fold and spot 44, 1.9-fold), and carbonic anhydrase (CA, spot 17, 3.1 fold, spot 27, 3.22 fold, and spot 40, 1.64-fold) were increased in relative abundance in 100 *μ*M As treatment except for transketolase (TK; spot 1) and RuBisCO activase (spot 22). Consistent with changes in Calvin cycle, three other important proteins involved in electron transport chain (fourth subgroup) such as, ATP synthase *α* subunit (spot 21, 2.7-fold), rieske protein (FeS, spot 24, 2.4-fold), and ferredoxin-NADP reductase (FNR, spots 8 and 38, 2.4- and 1.7-fold) were significantly enhanced only under 100 *μ*M As treatments. A marked increase in P protein subunit of glycine decarboxylase (spot 14, 2.0-fold in 100 *μ*M and 2.5-fold in 150 *μ*M) was found in As-stressed plants, this being more significant in case of 150 *μ*M As treatment. Cytoplasmic malate dehydrogenase (MDH, spot 39) depicted a significant upregulation (2.12-fold) under 150 *μ*M As but no change under 100 *μ*M As treatment. Two upregulated antioxidant proteins identified in this study, dehydroascorbate peroxidase (DHAR, spot 18) and ascorbate peroxidase (APX, spot 19), revealed significant accumulation of 3.4- and 3.1-fold in 100 *μ*M As treatments. Three proteins of the transcriptional regulator category, namely, glycine rich RNA binding protein (SGRP, spot 25), maturase K (spot26), and *LEAFY *like protein (spot 28), displayed similar abundance patterns in 100 and 150 *μ*M As treatments, with a slightly higher amount in 150 *μ*M As treatments. Four protein spots belonged to protein metabolism category. Of the four proteins, the rossmann NAD(P) fold (spot16) was found to be increased in both 100 and 150 *μ*M As treatments, whereas ribosomal L 12 1a (spot 13) revealed a 2.5-fold increase in 100 *μ*M As treatments and decrease in abundance in 150 *μ*M As treatments. In contrast to the above, Hsp 70 (spot 29) and chloroplast protease (spot 30) revealed decrease in relative abundance under 100 and 150 *μ*M As treatments. Besides this, two proteins were identified belonging to secondary metabolism category. The spot number 31 was identified as S-adenosyl methionine synthase (SAMS), a signaling protein involved in biosynthesis of homocysteine and ethylene, and revealed a 1.5-fold induction under 100 *μ*M As treatment. The other protein, chalcone synthase (spot 9), revealed a 2.2- and 2.3-fold increase in the amount of this protein under 100 and 150 *μ*M As treatments, respectively. Spot 35 was identified as a multidrug resistance-associated protein (MRP), a subclass of ATP-binding cassette (ABC) transporters depicted a 1.5-fold upregulation under 100 *μ*M As treatment. There were five protein spots that did not find match to any of the characterized proteins in the database and hence were named as UK (unknown).

### 3.8. Multivariate Statistical Analysis of Protein Abundance

**Figure 4:**
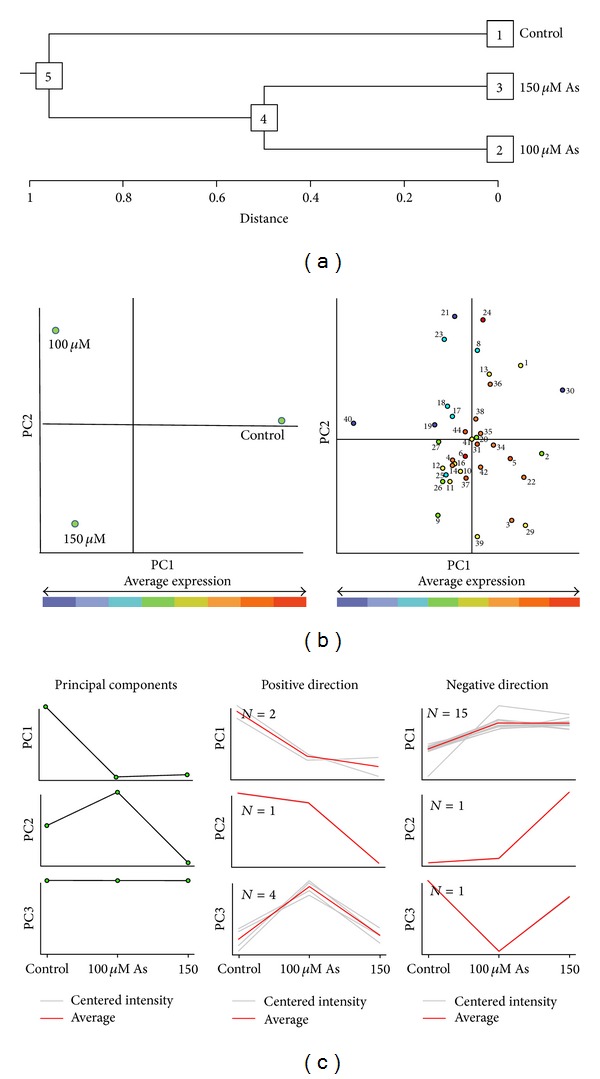
Multivariate analysis (PCA analysis) of control, 100 and 150 *μ*M As treatments using the ANOVA-based NIA array analysis tool. (a) Dendrogram showing hierarchical clustering of biological replicates of the control and As treatments. The expression clusters are numbered from 1 to 5. (b) Two-dimensional PCA biplots showing associations between experimental groups and protein spots generated by principal component analysis (PCA). Experimental groups (left) and protein spots (right) were plotted in the first two component spaces. Spots that are clustered together on the biplot should have similar expression profiles. (c) Protein spot abundance clustering based on PCA. For each PC, two clusters of proteins were identified that were positively and negatively correlated with the PC. Protein clustering was performed sequentially starting from the first PC. Proteins that were already clustered with a PC were not included in the clusters associated with subsequent PCs. The different colors of the spots represent the average expression patterns of proteins. Proteins are more expressed in those tissues which are located in the same area of the graph.

Principal component analysis (PCA) was done to obtain a more accurate grouping of the samples and to determine the most discriminant spots. In order to check out the reproducibility in the biological replicates, a hierarchical clustering was performed. Two main clusters were obtained in the dendrogram, namely, cluster 5 (control) and cluster 4 (100 and 150 *μ*M As treatments) (Figure 4(a)). This clustering indicated that 100 and 150 *μ*M As treatments had a somewhat similar protein abundance profiles but different from that of control. Moreover, the hierarchical clustering of biological repetitions confirmed that the data were reproducible for the experiments. PCA analysis indicated a distinct separation of 100 and 150 *μ*M As treatments (Figure 4(b)). Photosynthetic proteins were found in the categories of proteins correlated with PC1 (positive and negative direction) and PC3 (positive direction). Carbon metabolism proteins were found in the categories of proteins correlated with PC1 (negative direction), PC2 (positive and negative direction), and PC3 (positive direction). Proteins belonging to transcription as well as protein metabolism category were found in PC1 (positive and negative direction) and PC3 (positive direction). Defense and stress-related proteins were found in PC1 (negative direction). Transport proteins were found in PC3 (positive direction). A high proportion of photosynthetic, carbon metabolism and defense proteins were more abundant in 100 *μ*M As treatments with respect to the 150 *μ*M As treatments. The quantity of protein abundance change within a specific PC was calculated by the slope of regression of log-transformed protein abundance versus the corresponding eigenvector multiplied by the range of values within the eigenvector (Figure 4(c)).

### 3.9. RT-PCR Analysis of Isoprenoid/Artemisinin Biosynthetic Pathway Genes

**Figure 5:**
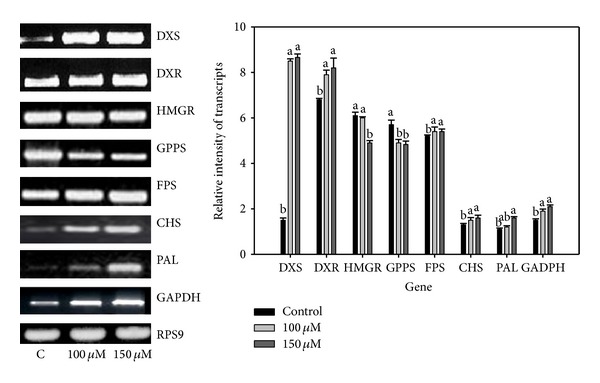
RT-PCR analysis of selected protein genes of flavonoid and artemisinin biosynthetic pathway. The experiment was repeated thrice. Ribosomal protein S9 (RPS9) was used as an internal control. DXS: 1-deoxy-d-xylulose 5-phosphate synthase; DXR: 1-deoxy-d-xylulose 5-phosphate reductoisomerase; HMGR: 3-hydroxy-3-methylglutaryl coenzyme A reductase; GPPS: geranyl pyrophosphate synthase; FPS: farnesyl diphosphate synthase; CHS: chalcone synthase; PAL: phenyl ammonia lyase; GAPDH: glyceraldehyde-3-phosphate dehydrogenase.

The expression profile of eight genes encoding enzymes of isoprenoid (MEP) and artemisinin biosynthetic pathways were monitored in the control and As treated plants (Figure 5). While all the six genes were upregulated in response to 100 *μ*M As treatment, a significant overexpression of 4.4- and 1.2-fold was observed for 1-deoxyxylulose-5-phosphate synthase (DXS) and 1-deoxy-D-xylulose 5-phosphate reductoisomerase (DXR), respectively. A similar pattern of gene expression was observed under 150 *μ*M As treatment. GAPDH also depicted an induction of 1.0- and 1.1-fold under 100 and 150 *μ*M As treatments, respectively. In contrast, geranyl pyrophosphate synthase (GPPS) and 3-hydroxy-3-methyl-glutaryl-CoA reductase (HMGR) showed minor down regulation in response to both As treatments (Figure 5).

## 4. Discussion

An integrated morphological, physiological, and proteomic approach has been used for the first time to investigate the mechanism of As tolerance of* A. annua*, a medicinal plant whose genome has not yet been sequenced. Although 46 proteins identified by MALDI-TOF/MS/MS and MS-BLAST analyses represent only a very small fraction of* A. annua* leaf proteome, some novel As stress responsive proteins appear involved in cellular homeostasis and survival of* A. annua* plants under As stress.

### 4.1. As Regulates Plant Architecture and Flowering Time in* A. annua*

The alterations observed in inflorescence branching patterns, vegetative axillary shoot development, and flowering time in As treated* A. annua* clearly indicate that these changes in plant form may arise, at least in part, due to change in expression pattern of conserved regulators or proteins. It is quite likely that the early onset of flowering and the increased number of florets and capitulum size in As treated* A. annua* were due to the significant accumulation of* LEAFY-* (LFY-) like protein, a plant-specific transcription factor involved in growth, development [53], and flowering in* Arabidopsis* [54], first time observed under As stress. Furthermore, the red coloration of the leaf apex and shoots of 150 *μ*M As treated* A. annua *plants could be due to transient accumulation of anthocyanin pigments below the leaf epidermis. This finding is supported by an increased accumulation of chalcone synthase, involved in the production of secondary metabolites and pigments such as flavonoids and anthocyanin as also observed in As-stressed plants [55].

### 4.2. Photosynthetic Apparatus under As Stress

The physiological traits monitored revealed contrastingly differences under 100 and 150 *μ*M As treatments. While PSI activity, transpiration rate, ATP, and NADPH contents of 100 *μ*M As treated* A. annua *plants showed an increase over the control values, a decrease for these parameters was recorded at 150 *μ*M As treatments. On the other hand, PSII activity registered decline under both the treatments, this being more pronounced at 150 *μ*M As treatments. The *F*_*v*_/*F*_*m*_ ratio, considered as indicator of the photochemical processes in photosystem 2 (PSII), depicted only a minor inhibition at 100 *μ*M but severe reduction under 150 *μ*M As treatments. Above findings were supported by the protein data having increased abundance of ATP synthase *α* subunit, rieske protein, and ferredoxin-NADP reductase (FNR) under 100 *μ*M As treatments, suggesting their likely role in As tolerance. Moreover, this enhancement unswervingly indicates operation of Fd-dependent cyclic electron transport (CET) in the test organism. In general, plants exposed to abiotic stresses show downregulation of linear electron flow (LEF) and activation of cyclic electron transport (CET) when linear electron flow becomes saturated [56, 57]. It could be anticipated that under 100 *μ*M As treatment the LEF is partially replaced by CET around PSI and the upregulated CET in turn provides energy to the Calvin cycle [58] thereby meeting the energy demand. Appreciable increase in NADPH content seems justified in view of its requirement as reducing equivalents during CO_2_ fixation on one hand and maintaining a ratio of reduced glutathione for scavenging ROS. By contrast, under 150 *μ*M As treatments, the down regulated FNR disrupts CET. The electrons so accumulated suppress the charge separation of PSII reaction centre leading to damage of PSII as attested by the results of decreased *F*_*v*_/*F*_*m*_ ratios and decreased ATP content (Table 2, Figure 7). Four proteins of chlorophyll a/b binding category (CP26 in PS II) displayed a mixed expression under 100 and 150 *μ*M As treatments. Altered levels of multiple forms of this protein have been reported in rice seedling under high temperature [59] and H_2_O_2_ stress [60]. Another protein (spot 11) identified as chloroplast PS I type III chlorophyll a/b binding protein was increased in 100 *μ*M As treatment. This may confer enhanced resistance of PSI as known for* Hordeum vulgare* against boron stress [61]. While (OECP2) protein spot 4 (intact) depicted increased relative abundance, the protein (spot 5) BLAST identified as oxygen-evolving complex protein (OEC1) was decreased under both treatments. OECP2 are manganese stabilizing proteins playing crucial roles in photosystem II stability [41], their decreased accumulation supports the damaged PSII under As treatments. A marked increase in P protein subunit of glycine associated with photorespiration was found in 150 mM As-stressed plants. Its overexpression stimulated photorespiration in* Anabaena* sp. under As stress [41]. These results clearly demonstrated that inhibition of photosynthetic rate in 150 *μ*M As treated* A. annua* was not the consequence of stomatal closure (reflected by high intercellular CO_2_ value) hence CO_2_ limitation may not be the real cause for reduced carbon assimilation rates. The decrease in assimilation rates could be due to low ATP and NADPH contents (Table 2) and consequent impairment of the photosynthesis [47, 62]. Furthermore, the photosynthetic rate is known to weaken in the presence of heavy metals due to alterations in the active site of rubisco subunits [63, 64]. By contrast, PSI complex machinery might be envisioned to support the damaged PSII and maintain NADPH pool in 100 *μ*M As treated plants as observed in As-tolerant cordgrass* Spartina densiflora* [65] and* Ricinus communis* [66].

### 4.3. Metabolic Networks under As Stress

Of the 15 identified proteins of the metabolic pathways like PLA, FBA class I, GAPDH, RuBP-5-P, TPI, and CA were increased in 100 *μ*M As treatment except for TK and RuBisCO activase. This finds support from Pandey et al. [41], where TK was decreased in abundance under As stress. Transketolase is a universal amphibolic enzyme catalyzing reactions in the Calvin cycle and the oxidative pentose pathway producing erythrose-4–phosphate, leading to phenylpropanoid metabolism. Thus, any change in its abundance is expected to affect both photosynthetic carbon assimilation and secondary metabolism in plants. Despite a decrease in the abundance of transketolase in As treated plants neither Calvin cycle nor secondary metabolite synthesis (artemisinin, carotenoid, etc.) reflected any downregulation. This indicates the likely complementation of its role by some other enzymes. Henkes et al. [67] reported that* Arabidopsis* TK shares 23% identity with DXS which depicted increased expression under decreased TK activity. Further, TK has been reported to be a DXS like protein [68] and hence speculated to play a role similar to transketolase. An* in silico* analysis of TK showed 38% homology with DXS in* A. annua *and RT-PCR analysis revealed a significant transcript in As treated* A. annua*, which together support our above speculation (Figure 5). Of the three protein species corresponding to chloroplastic CA, two appeared as products of the same gene (spots 17 and 27) whereas the third one (spot 40) was different. An increased accumulation of carbonic anhydrase may be required for interconversion of CO_2_ and HCO_3_^2^- and is thought to play a role in photosynthetic carbon assimilation as known in* Arabidopsis* [69] and other C3 plants [70, 71]. The increased abundance of various isoforms of FBA, GAPDH, and TPI clearly indicates the activation of the glycolytic pathway under As stress. FBA reversibly catalyzes the conversion of fructose 1, 6-bis phosphate (FBP) to GAP and dihydroxy acetone 3-phosphate (DHAP). During stress, GAP and FBP may be converted to glucose 6-phosphate for reentry into the PPP for NADPH synthesis. In the light of the reports of Pandey et al. [41], one is tempted to presume that enhanced expression of FBA maintained the glycolysis, PPP, and turnover rate of Calvin cycle. In contrast to the report of Ahsan et al. [20], significant accumulation of three isoforms of glyceraldehyde-3P-dehydrogenase (GAPDH) was noticed. GAPDH detoxifies As using arsenate instead of phosphate converting glyceraldehyde 3-phosphate into 1-arseno-3-phospho-glycerate. Enhanced expression of GAPDH has also been detected in* Pteris* under As stress [24] rice seedlings under drought, submergence, and ABA treatment [72]. These findings imply that increased accumulation of GAPDH may contribute to abiotic stress tolerance in plant. Of particular interest was the cytoplasmic MDH depicting significant accumulation under 150 *μ*M but no change under 100 *μ*M As treatment. This is conceivable because As stress leads to phosphate (Pi) starvation in plants (Supplementary Table S1). Though arsenic substitutes for phosphorus in the plants, it cannot function like phosphate, hence plants realize phosphate deficiency [73]. Under these situations, plants ought to cope up with Pi deficiency. Taken together, these results suggest an increased turnover of CO_2_ assimilating pathway and redox energy production in the 100 *μ*M As treated plants which are required for survival under downregulated photosynthetic rate. In contrast, decrease in carbon metabolism proteins associated with breakdown of energy generation results in failure of the plants to cope up with 150 *μ*M As toxicity.

### 4.4. Regulation of Antioxidant Machinery, Protein Metabolism, and Transport under As Stress

Two antioxidant proteins identified in this study include dehydroascorbate peroxidase and ascorbate peroxidase in 100 *μ*M As treatments. While DHAR (spot 18) has been reported to offer tolerance against oxidative stress [74], APX catalyses the reduction of H_2_O_2_ to water in ascorbate-glutathione cycle [75]. Increased abundance of APX under stress is nicely documented in earlier proteomic works [76, 77]. It is quite likely that the upregulated ascorbate-glutathione cycle regulates excess ROS generated at PSII thereby offering tolerance in 100 *μ*M As treated plants. This is in accordance with the findings of Rai et al. [11]. Three proteins belonging to transcriptional regulator category, namely, glycine rich RNA binding protein, maturase K, and *LEAFY* like  protein displayed analogous abundance in 100 and 150 *μ*M As treatments, with a slightly higher level in 150 *μ*M As. Glycine rich RNA binding proteins are implicated in osmotic, cold stress, and RNA splicing activity [78], and maturase K functions like a ribosomal protein with RNA chaperone activity [79]. Increased accumulation of these proteins suggest their conceivable role in overcoming the As stress. Another interesting observation was upregulated protein synthesis in* A. annua* under As stress. Of the four proteins belonging to the protein metabolism category, the rossmann NAD(P) fold, a structural motif found in nucleotide binding proteins [80], was increased under both As treatments, whereas ribosomal L 12 was increased in 100 *μ*M but decreased in 150 *μ*M As treatments. In contrast to the above, two molecular chaperones, Hsp70 and chloroplast protease were decreased under both As treatments. Significant down regulation of Hsp70 suggests it to be the key target under As stress as observed in* Poplar *trees under Cd stress [17] and needs further investigation.

**Figure 6:**
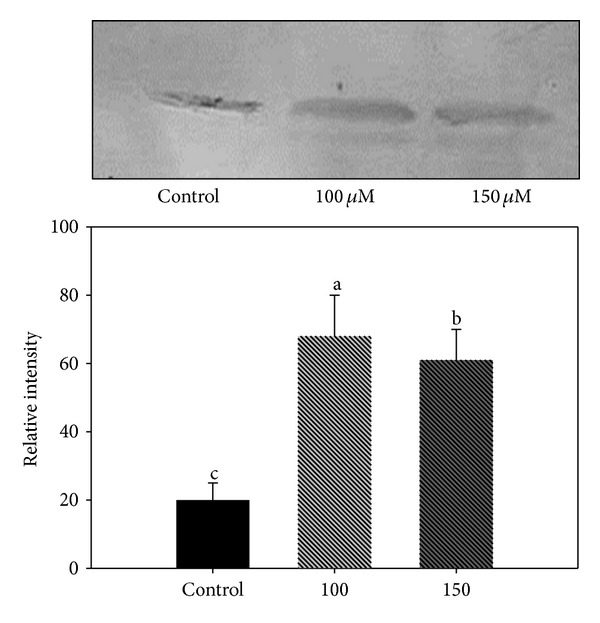
Western blot of PCS protein after treatment with 100 and 150 *μ*M As concentrations. Protein separated by SDS-PAGE, electroblotted onto a PVDF membrane, and cross-reacted with primary antibody (anti-PCS antibody).

Of particular interest are the multidrug resistance-associated proteins (MRPs), a subclass of ATP-binding cassette (ABC) transporters with a hypothetical role in As extrusion in plant. Our previous finding [11] impelled to check the role of* pcs* gene in As detoxification. The upregulated (1.5-fold) mdr-like ABC transporter and* pcs* gene (as confirmed by Western blot analysis, Figure 6) under 100 *μ*M As suggest that detoxification was brought about by reduction of As(V) to As(III) and sequestration with phytochelatin. The increased abundance of (ABC) transporters has also been reported in* Comamonas* sp. under As stress [81]. Another interesting observation was the increased accumulation of S-adenosyl methionine synthase (SAMS), known to act as a signaling molecule and protects cells from arsenic stress by forming S-adenosyl-methionine [20]. Furthermore, in the light of the report of Noriega et al. [82] that SAM protects cells from deleterious effects of cadmium, its increased accumulation under 100 *μ*M As treatment seems justified.

### 4.5. Hypothetical Model Depicting As Tolerance and Interaction between Primary and Secondary Metabolism in* A. annua*

**Figure 7:**
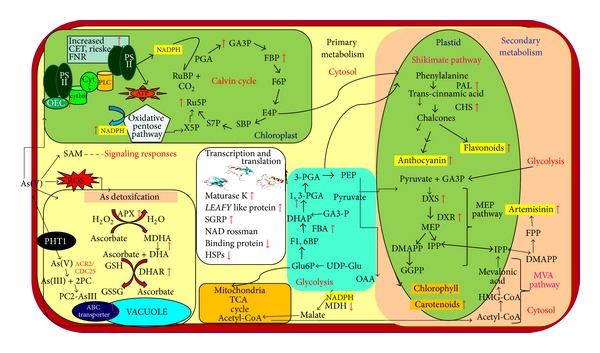
Hypothetical model depicting As tolerance and interactive protein network between primary and secondary metabolism in* Artemisia annua.*

Taking recourse to 39 As-responsive proteins as well as the data available in the literature, a protein network model has been proposed. This network consists of several functional components, including ROS production and scavenging, electron transport chain, protein metabolism, photosynthesis, energy supply, and biosynthesis of signaling molecules (Figure 7). The model clearly shows that As(V) may be taken up by the test plants through phosphate transporter Pht1 and reduced to arsenite As(III) by arsenate reductase (ACR2/CDC 25) in the cell. As(III) is rapidly complexed with soluble thiols and phytochelatins. Upon binding to phytochelatins (PCs), PC2-As(III) complexes are deposited into the vacuole by ABC transporters. This is clearly reflected by increased phytochelatin content and transcript of* pcs *gene under As stress. To cope up with the As stress,* A. annua *plants in turn activate their antioxidative and photosynthetic machineries. Two groups of proteins identified in multiple isoforms are chlorophyll a, b binding protein as well as OEC of PSII. OEC appears to be involved in the oxidation of water molecules to form molecular oxygen and ATP production via proton pumps. The excess electrons are transferred to oxygen at PSI or via Mehler reaction. In order to protect PSII from ROS, As-stressed plants augment excess electron removal by activating ascorbate-glutathione cycle such as APX and DHAR. An increased abundance of FBA, Ru5P, ALD, and CA (Table 3) points not only enhanced turnover of CO_2_, assimilating pathway but also augmentation in ATP and NADPH synthesis. This was clearly reflected by an increased cyclic electron flow around PSI through increased accumulation of Fd-NADP reductase, rieske-Fes, and ATP synthase. The increased ATP and redox energy support enhanced CO_2_ assimilation. The enhanced glycolysis provides not only reducing power required for As detoxification and repair but also precursors for biosynthesis of aromatic amino acid, flavonoid, and anthocyanin (via shikimate pathway) and artemisinin (via MEP pathway). Interestingly, two intermediates of glycolysis, GAPDH and pyruvate, are direct substrates of the DXP synthase. The upregulation of GAPDH on 2DE gel indicates persistent triose supply for the synthesis of artemisinin, anthocyanin, and carotenoids. Furthermore, the upregulated Calvin cycle and PPP produce erythrose-4-phosphate, a precursor for the shikimate pathway leading to phenylpropanoid metabolism. The rapid consumption of glyceraldehyde-3-P and other primary metabolites requiring continuous replenishment of carbon intermediates may in turn be provided by ribulose-5-P (with 2-fold accumulation on 2DE gel). Presence of only one protein (CHS) belonging to a flavonoid biosynthetic pathway may be due probably to limited number of protein spots analyzed. The network connection between primary and secondary metabolism of* A. annua* measured by the transcript of enzymes of flavonoid and artemisinin biosynthetic pathway such as DXS, DXR, FPS, HMGR, GPPS, CHS, and PAL indicated their fine regulatory network.

## 5. Conclusions

Of the different proteins identified in this study, proteins of photosynthesis and carbon metabolism appear as possible candidates for augmenting As tolerance in* A. annua*, although the exact mechanism of tolerance remains to be investigated. Our results also suggest that ABC-transporter like protein may be involved in As sequestration into vacuoles as the final detoxification step. Data obtained in this study provide clues to the molecular aspects of As tolerance, as well as in development of As resistant crop varieties through plant breeding and biotechnological interventions.

## Supplementary Material

Table S1: Total As and phosphate content in plant tissues.
Table S1: Total As and phosphate content in plant tissues.
Table S3: Description of the features of the 2-DE experiment
Table S4: Analysis of Variance of 39 differentially expressed protein spots of 100 & 150*μ*M As treatments before and after transformation of data (log transformation).
Table S5: List of peptide sequence and charge of protein spots.
Table S6: BLAST P analysis results.
Fig. S1: Relative intensity of identified proteins.
Fig. S2: Functional classification of identified proteins.
Fig. S3: Expression pattern of selected proteins of C, control and; 100, 150 *μ*M As treatments.

## Figures and Tables

**Table 1:** Effect of As on various morphological and physiological parameters including, plant height, flowering time, number of inflorescences/plant, number of branches/inflorescence, number of capitula/inflorescence, capitula size, florets distance in whorl, number of florets/capitulum, number of oil glands, and artemisinin and anthocyanin contents in *A*. *annua* plants. Measurements were conducted under natural conditions when plants were 8-month old.

Characters	Control		100 *µ*M		150 *µ*M	
Mature plant height (m)	1.4 ± 0.04^a^		1.2 ± 0.02^a^		1.1 ± 0.09^a^	
Time of first flower	30-31 weeks		28-29 weeks		22−24 weeks	
Number of inflorescence/plant	28 ± 1.02^a^		27 ± 1.34^ab^		24 ± 0.98^c^	
Number of branches/inflorescence	23 ± 1.1^a^		21 ± 1.1^a^		18 ± 1.4^b^	
Number of capitula/inflorescence	20 ± 1.02^b^		32 ± 0.98^a^		33 ± 1.04^a^	
Capitula size (mm)	4.3 ± 0.95^c^		5.1 ± 0.76^b^		5.4 ± 0.01^a^	
Distance of florets in involucre whorl (mm)	1.1 ± 0.004^a^		1.0 ± 0.01^a^		0.8 ± 0.003^b^	
Florets/capitulum	Ray = 13	Disc = 35	Ray = 16	Disc = 45	Ray = 17	Disc = 55
Artemisinin content (mg/100 g DW)	0.39 ± 0.02^a^		0.46 ± 1.02^a^		0.52 ± 0.02^a^	
Number of oil glands/florets	5 ± 0.05^c^		7 ± 0.09^b^		9.9 ± 0.01^a^	
Anthocyanin content (*µ*mol g^−1^ FW)	0.06 ± 0.01^a^		0.07 ± 0.06^a^		0.12 ± 0.09^a^	
Biomass (DW g plant^−1^)	241 ± 4.5^b^		267.0 ± 5.2^a^		221 ± 5.0^c^	

All values are mean ± SD (*n* = 3). Different letters show significantly different values (*P* < 0.05, DMRT).

**Table 2:** Photosynthetic rate (*P*_*s*_), stomatal conductance (*g*_*s*_), intercellular CO_2_ (*C*_*i*_), transpiration rate (*E*), water use efficiency (WUE), *F*_*v*_/*F*_*m*_ (the maximal photochemical efficiency), photosystem I activity (PS-I), photosystem II activity (PS-II), whole chain electron transport chain, ATP and NADPH contents of control, and As treated *A*. *annua* plants. Measurements were conducted when plants were 8-month old.

Physiological parameters	Control	100 µM	150 µM
*P*_*s*_ (*µ*mol CO_2_ m^−2^ s^−1^)	31.7 ± 0.43^a^	30.0 ± 0.78^b^	23.5 ± 0.99^c^
*g*_*s*_ (mol H_2_O m^−2^ s^−1^)	0.44 ± 0.04^a^	0.36 ± 0.07^a^	0.34 ± 0.08^a^
*C*_*i*_ (ppm)	201.0 ± 8.9^a^	189.0 ± 9.7^a^	212.0 ± 5.7^a^
*E* (mmol H_2_O m^−2^ s^−1^)	6.1 ± 0.45^a^	6.08 ± 0.67^a^	6.02 ± 0.54^a^
WUE (µmol mol^−1^)	3.2 ± 0.02^b^	5.1 ± 0.82^a^	4.9 ± 1.1^a^
*F*_*v*_/*F*_*m*_	0.82 ± 0.01^a^	0.79 ± 0.02^a^	0.72 ± 0.04^b^
PS-I activity (*µ*mol O_2_ consumed mg^−1^Chl h^−1^)	250 ± 9.01^c^	375 ± 11.0^a^	279 ± 10.0^b^
PS-II activity (µmol O_2_ evolved mg^−1^Chl h^−1^)	225 ± 11.0^a^	199 ± 12.0^b^	154 ± 12.0^c^
Whole chain (µmol O_2_ evolved mg^−1^Chl h^−1^)	148 ± 12^a^	149 ± 22.3^a^	109 ± 11^b^
ATP (µmol g^−1^ FW)	0.37 ± 0.07^a^	0.40 ± 0.12^a^	0.29 ± 0.07^a^
NADPH (nmol g^−1^ FW)	5.17 ± 0.61^b^	7.12 ± 0.82^a^	3.91 ± 0.71^b^

All values are mean ± SD (*n* = 6). Different letters show significantly different values (*P* < 0.05, DMRT).

**Table 3:** Arsenic-induced differentially expressed proteins in *Artemisia annua* identified by MALDI-MS/MS analysis.

Spot No.	Protein name	Plant species/accession number	Mr/p*I* Theoretical^a^	Mr/p*I* Experimental^b^	Score^c^		Matched peptides^d^	Coverage (%)^e^	Cellular location	Blast P results^f^	Fold change^g^	
					Protein	Peptide						
	Energy metabolism (primary carbon metabolism)											
1	Transketolase	*Spinacia oleracea*/gi∣68052991	80.7/6.2	66.0/5.9	87	50	2	12	Chloroplast	—	−1.89^b^	−2.10^a^
6	Glyceraldehyde-3-phosphate dehydrogenase	*Mikaniamicrantha*/gi∣269856436	37.0/8.5	32.0/6.8	96	63	2	9	Cytosol	—	1.85^a^	1.87^a^
37	Glyceraldehyde-3-phosphate dehydrogenase, putative	*Ricinus communis/*gi∣255539282	49.1/7.5	36.0/6.0	257	132	4	9	Cytosol	—	1.60^b^	2.04^a^
23	Unknown	*Glycine max/*gi∣255641007	48.8/6.7	29.0/4.1	257	136	4	11	Cytosol	Glyceraldehyde-3-phosphate dehydrogenase	4.51^a^	1.70^b^
22	Ribulose-1,5-bisphosphate carboxylase activase	*Glycine max/*gi∣358249078	48.8/6.2	40.0/4.9	418	122	4	13	Chloroplast	—	−1.55^a^	−1.72^a^
36	Plastidic aldolases	*Solanum tuberosum*/gi∣1781348	38.6/5.8	32.0/5.8	303	113	5	28	Chloroplast	—	1.50^a^	−1.08^b^
12	Plastidic aldolases	*Solanum tuberosum*/gi∣1781348	38.6/5.8	20.0/5.5	279	103	3	12	Chloroplast		2.69^a^	1.96^a^
17	Carbonic anhydrase	*Flaveria bidentis/gi*∣*1089983 *	36.0./5.8	26.0/6.9	128	87	2	13	Chloroplast	—	3.10^a^	2.00^b^
27	Carbonic anhydrase 3	*Flaveria bidentisi*/gi∣1089983	36.0/5.8	29.0/6.5	209	111	4	9	Chloroplast	—	3.22^a^	2.86^b^
40	Carbonic anhydrase	*Dimocarpus longan*/gi∣339958979	35.2/6.5	24.0/6.4	128	87	2	10	Chloroplast	—	4.4^a^	2.3^b^
30	ATP dependent Chloroplast protease putative	*Ricinus communis/*gi∣255537123	30.9/8.7	58.0/5.4	90	90	1	5	Chloroplast	—	−3.02^a^	−3.63^a^
34	Fuctose-bis-phospahte aldolase,	*Spinacia oleracea/*gi∣22633	42.7/7.5	27.0/4.5	93	39	3	10	Chloroplast	—	2.12^a^	1.04^b^
39	Malate dehydrogenase	*Solanum tuberosum/*gi∣21388544	36.4/8.8	34.0/6.4	248	157	3	16	Mitochondria	—	−0.75^b^	2.12^a^
41	Ribose-5-phosphate-isomerase	*Spinacia oleracea*/gi∣18654317	30.9/6.5	25.0/6.1	69	69	1	10	Chloroplast	—	1.72^a^	1.54^a^
44	Triosephosphate isomerase	*Medicago tranculata*/gi∣1351282	27.7/6.5	25.0/4.9	227	130	3	8	Cytosolic	—	1.29^a^	1.50^a^
42	Triosephosphate isomerase	*Medicago tranculata*/gi∣1351282	27.7/6.5	26.0/5.6	129	89	2	11	Cytosolic	—	1.49^a^	1.50^a^
4	PS II PsbP protein (Oygen Evolving enhancer 2)	*Pisum sativum/*gi∣131390	28.2/8.2	22.0/6.4	138	54	6	9	Chloroplast	—	2.24^a^	2.46^a^
3	Photosynthetic electron transfer-like protein	*Panax ginseng/*gi∣89475526	19.9/5.7	20.0/6.1	132	94	2	14	Chloroplast		−1.04^b^	1.39^a^
5	Unnamed protein product	*Spinacia oleracea/*gi∣21283	31.9/7.6	28.0/5.2	252	76	3	20	Chloroplast	PSII-O, photosystem II, oxygen evolving enhancer	1.39^a^	1.49^a^
8	Ferredoxin NADP+ oxidoreductase	*Solanum peruvianum/*gi∣61969078	5.4/7.7	22.0/6.8	247	112	4	7	Chloroplast	—	2.41^a^	−1.50^b^
38	Ferredoxin NADP+ oxidoreductase	*Solanum peruvianum/*gi∣61969078	35.4/7.7	28.0/6.0	358	146	5	16	Chloroplast	—	1.70^a^	1.18^b^
2	Chlorophyll a, b, binding protein	*Solanum lycopersicum*/gi∣115813	29.3/8.9	23.0/5.9	63	63	1	10	Chloroplast	—	−1.93^b^	−1.50^a^
11	Chloroplast PSI Type III	*Helianthus annuus*/gi∣159138839	13.8/4.5	24.0/5.8	113	71	2	19	Chloroplast	—	2.83^b^	2.36^a^
10	Chlorophyll a, b, binding protein	*Pisum sativum/*gi∣20671	29.3/5.2	23.0/5.9	86	44	2	14	Chloroplast	—	1.99^b^	2.17^a^
20	Unknown protein	*Medicago tranculata*/gi∣217071344	31.2/6.5	38.0/4.3	149	54	3	10	Chloroplast	Chlorophyll a, b, binding protein	3.12^a^	1.35^b^
21	ATP synthase *β* chain	*Viburnum opulus*/gi∣7688419	22.7/4.3	45.0/5.2	55	16	2	76	Chloroplast	—	2.68^a^	−0.56^b^
24	Rieske-FeS protein	*Arabidopsis thaliana/*gi∣9843639	24.6/8.8	15.0/5.1	49	49	1	5	Chloroplast	—	2.35^a^	−1.06^b^

Energy metabolism (photorespiration)												
14	P protein	*Flaveria pringlei*/gi∣438003	11.4/6.5	8.0/4.5	79	41	2	10	Chloroplast	—	2.24^a^	2.46^a^

ROS scavenging and defence												
18	Dehydro ascorbate reductase	*Lotus japonicas/*gi∣66732627	29.1/7.7	26.0/6.0	83	83	1	4	Cytosol	—	3.07^a^	1.98^b^
19	Ascorbate peroxidase-2-like protein	*Tragopogan dubius/*gi∣290796648	13.1/4.5	27.0/5.3	91	91	1	20	Cytosol	—	3.44^a^	2.67^b^

Transcriptional Regulator												
25	SGRP glycine rich binding protein	*Daucus carota*/gi∣544426	15.7/5.3	15.0/5.3	157	107	3	25	Cytoplasm	—	2.83^b^	3.00^a^
26	Maturase K	*Prinsepia uniflora*/gi∣290583766	32.2/9.0	26.0/5.8	56	47	1	13	Cytoplasm	—	2.90^b^	3.16^a^
28	*LEAFY* like protein	*Mimulus guttatus*/gi∣14573451	9.1/9.6	25.0/4.8	20	20	1	16	Cytoplasm	—	2.32^a^	2.38^a^

Protein metabolism												
13	Ribosomal L12 1a	*Nicotiana tabacum/*gi∣20020	20.3/6.04	20.0/4.6	102	102	1	7	Cytoplasm	—	2.45^b^	−1.25^a^
16	NAD(P)-rossaman binding protein	*Arabidopsis thaliana/*gi∣18404496	34.9/8.37	15.0/6.3	114	61	2	10	Cytoplasm	—	2.19^a^	2.29^a^
29	Chloroplast heat shock protein 70-1	*Ipomoea nil*/gi∣166919370	74.4/5.1	63.0/4.8	308	124	4	10	Cytoplasm	—	−2.43^a^	−1.79^b^

Secondary metabolism												
9	Chalcone synthase	*Leibnitzia anandria*/gi∣1403057	25.4/5.4	25.0/6.5	50	50	1	8	Cytoplasm	—	2.24^b^	2.30^a^
31	S-adenosyl methionine synthase	*Arabidopsis thaliana/*gi∣15228048	42.4/5.7	38.0/5.8	281	93	5	16	Cytoplasm	—	1.61^a^	1.41^a^

Transport protein												
35	ABC transporter like protein	*Arabidopsis thaliana/*gi∣224090097	66.6/9.6	29.0/5.5	27	27	1	10	Cytoplasm	—	1.50^a^	1.28^a^

Unknown proteins												
32	Unknown protein	*Populus trichorpa/*gi∣224130876	83.0/5.9	97.0/6.2	26	26	1	2	—	Unknown protein	−1.9^a^	−1.7^a^
33	Hypothetical protein	*Vitis vinifera/*gi∣147845220	60.5/8.68	55.0/5.8	38	38	1	5	—	Hypothetical protein	1.5^b^	1.9^a^
43	Unknown protein	*Zea mays*/gi∣302844664	17.0/7.8	24.0/5.4	30	30	1	4	—	Unknown protein	1.99^a^	1.67^b^
45	Hypothetical protein	*Sorghum bicolor*/gi∣242061954	46.2/6.5	22.0/4.7	43	29	3	1	—	Hypothetical protein	1.67^b^	1.80^a^
46	Unknown protein	*Vitis vinifera/*gi∣296081316	47.9/9.4	21.0/4.3	33	33	1	2	—	Unknown protein		

^a^Theoretical mass (kDa) and p*I* of identified proteins were calculated consulting databases NCBI nr and Swissprot.

^b^Experimental mass (kDa) and p*I* of identified proteins.

^c^Score: the protein score is derived from the ions scores from an MS/MS search.

^d^Matched peptides derived from MS/MS data.

^e^Coverage (%): it is the percent of the residues in each protein sequence that have been identified.

^f^Blast P result similar to the result as obtained in NCBInr database is indicated by (—).

^g^Fold change is calculated as a ratio of the averaged means of normalized spot volumes of control and the treatments.

## Abbreviations

ANOVA: Analysis of variance
CBB: Coomassie Brilliant Blue
CHAPS: 3-(3-Cholamidopropyl) dimethylammonio-1-propane sulfonate
FBA: Fructose bisphosphatase aldolase class I
GAPDH: Glyceraldehyde-3-phosphate dehydrogenase
Ru-5-P: Ribulose-5-phosphate, 3-epimerase
CA: Carbonic anhydrase
TK: Transketolase
MDH: Malate dehydrogenase
TPI: Triose phosphate isomerase
GRP: Glycine–rich RNA binding protein
DMAPP: Dimethylallyl diphosphate
DXS: 1-Deoxy-d-xylulose 5-phosphate synthase
DXR: 1-Deoxy-d-xylulose 5-phosphate reductoisomerase
FPS: Farnesyl diphosphate synthase
HMGR: 3-Hydroxy-3-methylglutaryl coenzyme A reductase
GPPS: Geranyl pyrophosphate synthase
IPP: isopentenyl diphosphate
MEP: 2-C-methyl-d-erythritol-4-phosphate
MVA: Mevalonic acid
CHS: Chalcone synthase
PAL: Phenyl ammonia lyase
PCA: Principal component analysis
FDR: False discovery rate.
