# Supplementary material for: Enhanced Photosynthesis and Carbon Metabolism Favor Arsenic Tolerance in Artemisia annua, a Medicinal Plant as Revealed by Homology-Based Proteomics
Source: Int J Proteomics. 2014 Apr 29;2014:163962. doi: 10.1155/2014/163962 (PMC4020366; doi:10.1155/2014/163962)
Supplement: Supplementary file 1 — Table S1: Total As and phosphate content in plant tissues. Table S1: Total As and phosphate content in plant tissues. Table S3: Description of the features of the 2-DE experiment Table S4: Analysis of Variance of 39 differentially expressed protein spots of 100 & 150μM As treatments before and after transformation of data (log transformation). Table S5: List of peptide sequence and charge of protein spots. Table S6: BLAST P analysis results. Fig. S1: Relative intensity of identified proteins. Fig. S2: Functional classification of identified proteins. Fig. S3: Expression pattern of selected proteins of C, control and; 100, 150 μM As treatments. [file 163962.f1.pdf]

Enhanced photosynthesis and Carbon metabolism favour Arsenic tolerance in *Artemisia annua* as revealed by homology based Proteomics

Rashmi Rai[1], Sarita Pandey[2], Alok Kumar Shrivastava [2] , Shashi Pandey Rai[1] *

Table S1 Total As and phosphate content in plant tissues

Arsenic (µM)	Total As content in plant tissues (µg g^{-1} DW)		Total Phosphate content in plant tissues (µg g^{-1} dw)
	Shoot	Root	
0	5.01 ± 0.098 [a]	1.67 ± 0.007 [a]	20.09±0.02[c]
100	91.72 ± 0.052 [b]	45.04 ± 0.008 [b]	15.09±0.03 [b]
150	131.09 ± 0.001 [c]	60.01 ± 0.023 [c]	12.01±0.002 [a]

Table S2. Primers used for RT-PCR analysis

Gene	Accession No.	Primer Sequence (5'-3')
DXS	AF182287	F: ATGGATCTCCGTCGTAAACTGCCA<br>R: GAGCGTTTGAGCCTGGTGATTCTA
DXR	AF182286	F: CTAGGATCCATGTCTTTGAACACCCTT<br>R:CTACTCGAGTCATACTAGTGCCGGAGT
HMGR	AF142473	F: CTAGGATCCATGGCACTTTCTGCATTT<br>R:CTACTCGAGCTATGACATGACCTCCAA
GPPS	Gi2578021	F: GGGATCCATGCTCTGTAAGATTATTAT<br>R: CGGAATTCATGGTGCAACAGTCAG
FPS	AF112881	F:GATTACCAACTCACAAAACT<br>R:GCTCAGAACCAACTATGATA
CHS	GQ468548	F: TGGGTAGCATCGATCTGAAAT<br>R: TTTGCCTCTTGTAGATTTTACC
PAL	NA	F:TGTAACTTCCAAGGAACCCC<br>R:CCACCAGAAAGATTTGAAGGCA

GAPDH	NA	F: TGGCTACTGCTGTT<br>R:TTAGTATTCAACGTT
RPS9 [a]	Gi145337556	F: GCGTTTGGATGCTGACTTGA<br>R:GGCGCTCAAGGAAGTTCTCTAC

[a] control primer

NA not applicable

Table S3. Description of the features of the 2-DE experiment

**Control + Treatments**	**Protein yield (mg g^{-1} FW)**	**No. of spots (mean ± SD)**	**No. of variable spots (control as reference)**	**Number of differential spots**		
				Total	Quantitative	Qualitative
Control	42±1.2	391±35	-	-	-	-
100 µM	37±1.8	412±41	46	46	41 (35 [a]) (6 [b])	3 [c] 2 [d]
150 µM	28± 1.4	447±27	51	46	40 (28[a]) (12 [b])	2 [e] , 4[f]

*The following criteria were used to consider a spot as being variable:
i) consistently present or absent in all the three replicates;
ii) display ≥ 1.5 fold change (ratio) between control and the treatments (P≤0.05).
(a) spots up accumulated in either 100 or 150 µM As treatments
(b) spots down accumulated in either 100 or 150 µM As treatments
(c) spots appeared in 100 µM (spot 8, 9, 40)
(d) spot non detected in 100 µM As treatment (spot 32,46)
(e) spots appeared in 150 µM (spot 9, 39)
(f) spot non detected in 150 µM As treatment (spot 29, 30, 32,46)

Table S4: Analysis of Variance of 39 differentially expressed protein spots of 100 uM & 150uM As treatments before and after transformation of data (log transformation)

	100uM As treatments						150uM As treatments					
	Before transformation			After transformation			Before transformation			After transformation		
Spot No	Mean	Std. Dev.	% CV	Mean	Std. Dev.	% CV	Mean	Std. Dev.	% CV	Mean	Std. Dev.	% CV
1	152	10.2	6.710526	2.18119	0.023836	1.09	75	5.6	7.466667	1.874252	0.026532	1.42
2	69	9.301613	13.4806	1.836186	0.048237	2.63	72	4.70319	6.532209	1.856712	0.023249	1.25
3	100.0333	8.051294	8.048611	1.999202	0.028646	1.43	180.2333	11.45178	6.353865	2.25525	0.022543	1.00
4	202	15.4	7.623762	2.304508	0.027093	1.18	212	14.7	6.933962	2.325638	0.024632	1.06
5	176.1	15.25025	8.659992	2.24467	0.030782	1.37	188.9667	12.55003	6.641401	2.275745	0.023593	1.04
6	299	4.8	1.605351	2.475634	0.005693	0.23	304	5.8	1.907895	2.482821	0.006766	0.27
8	136.9	6.701492	4.895173	2.136051	0.017553	0.82	57.96667	6.018582	10.38283	1.761568	0.037687	2.14
9	103.4	14.20845	13.74125	2.011847	0.047912	2.38	169.4	11.60517	6.85075	2.228234	0.024292	1.09
10	149.2333	7.956339	5.331476	2.173453	0.01896	0.87	172.2667	8.503137	4.936031	2.235849	0.017499	0.78
11	160.2667	12.16237	7.588835	2.204004	0.027048	1.23	200.5333	12.90633	6.436002	2.301584	0.022897	0.99
12	173.4667	8.681782	5.00487	2.238848	0.017941	0.80	192.8	7.968061	4.132812	2.284858	0.014731	0.64
13	189.2667	8.105142	4.282393	2.276808	0.015216	0.67	98.73333	8.450049	8.558456	1.9934	0.030424	1.53
14	205.9667	8.312841	4.036013	2.31356	0.014348	0.62	226.1667	7.626489	3.372066	2.354263	0.011991	0.51
16	159.5	8.550439	5.360777	2.202344	0.019021	0.86	172.2	11.70128	6.79517	2.235364	0.024116	1.08
17	109.7	7.554469	6.88648	2.039517	0.024513	1.20	79.4	2.511971	3.163692	1.899677	0.01112	0.59
18	127.9	7.037755	5.502545	2.106426	0.019707	0.94	84.83333	4.384442	5.168301	1.928186	0.018115	0.94
19	93.26667	8.003333	8.581129	1.96866	0.030447	1.55	71.73333	4.461315	6.219306	1.855157	0.02217	1.20
20	113	10.14889	8.98132	2.051886	0.032351	1.58	99.66667	9.609024	9.641161	1.99722	0.033886	1.70
21	85.73333	4.518112	5.269959	1.932745	0.018782	0.97	26.7	1.126943	4.220759	1.426257	0.014795	1.04
22	112.4333	5.501212	4.892866	2.050549	0.01735	0.85	143	2.645751	1.850176	2.155287	0.006532	0.30
23	149	5.8	3.892617	2.172967	0.013811	0.64	56.13333	3.950105	7.037005	1.748503	0.024989	1.43
24	603.4	11.78474	1.953055	2.78055	0.006907	0.25	197.9333	4.235957	2.140093	2.296452	0.007632	0.33
25	98.93333	8.700192	8.793994	1.994218	0.03129	1.57	116.3	8.970507	7.713248	2.064711	0.027535	1.33
26	109.0333	1.850225	1.696935	2.037518	0.006017	0.30	135.9	6.274552	4.617036	2.132916	0.016177	0.76
27	142.2333	7.152855	5.028958	2.152635	0.017828	0.83	126.6333	5.504846	4.347075	2.102274	0.01545	0.73
28	107.3333	4.02782	3.752627	2.030528	0.013453	0.66	112.1667	2.254625	2.010067	2.049805	0.007121	0.35
29	76.53333	2.557994	3.342327	1.883689	0.01183	0.63	146.0667	5.254839	3.597562	2.164361	0.012895	0.60

30	31.7	5.350701	16.87918	1.496888	0.060359	4.03	19.93333	4.673685	23.44658	1.291878	0.081082	6.28
31	220	11.78983	5.359012	2.342012	0.01884	0.80	205.2333	9.211044	4.488084	2.311961	0.015731	0.68
34	167.4333	51.04237	30.48519	2.207999	0.121866	5.52	153.3333	6.416645	4.184768	2.185384	0.01481	0.68
35	220.1	10.21323	4.640267	2.342311	0.016354	0.70	186.7667	7.139561	3.822717	2.271089	0.013516	0.60
36	197.9667	6.864644	3.467576	2.296419	0.012217	0.53	112.2	4.794789	4.27343	2.049732	0.014987	0.73
37	244.9	11.15034	4.553016	2.388688	0.016163	0.68	301	6.9	2.292359	2.47849	0.00813	0.33
38	194.8	5.574944	2.861881	2.289471	0.010094	0.44	146.4	5.047772	3.447932	2.16537	0.012151	0.56
39	101.3	3.858756	3.809236	2.005399	0.013545	0.68	204.2667	3.308071	1.619486	2.31016	0.005738	0.25
40	175.0667	3.787259	2.163324	2.243136	0.007638	0.34	125.7333	4.716284	3.751021	2.099249	0.013204	0.63
41	171.9667	6.115826	3.556402	2.235263	0.012491	0.56	153.1	4.660472	3.044071	2.184842	0.01073	0.49
42	188.1333	6.900966	3.668125	2.274271	0.013007	0.57	213.5667	6.061628	2.838284	2.329417	0.010083	0.43
44	269.9667	2.779089	1.029419	2.431295	0.003642	0.15	224.9667	7.150058	3.178275	2.351972	0.011276	0.48

Table S5: Peptide sequence, and charge of protein spots

Spot no.	Protein name	Peptide sequence	Charge
1	Transketolase	K.ALPTYTPETPGDATR.N	+1
2	Chlorophylll a, b, binding protein	K.WLAYGEVINGR.F	+1
3	Photosynthetic electron transfer-like protein	K.VVFVPWTETDFR.T<br>K.FMCPCHGSQYNNQGK.V	+1
4	PS II PsbP protein (Oygen Evolving enhancer 2)	R.EFPGQVLR.Y<br>K.EREFPGQVLR.Y<br>K.SITDYGSPEEFLSK.V	+1
5	PSII-O, photosystem II, oxygen evolving enhancer 1	R.VPFLFTIK.Q<br>R.GGSTGYDNAVALPAGGR.G<br>K.DGIDYAAVTVQLPGGER.V	+1
6	Glyceraldehyde-3-phosphate dehydrogenase	K.LVSWYDNEWGYSSR.V<br>K.GILGYVDEDVVSTDFLGDSR.S	+1
8	Ferredoxin NADP+ oxidoreductase	K.EPYVGR.C<br>R.LYSIASSALGDFGDSK.T<br>K.DPNATVIMLATGTGIAPFR.S<br>K.DPNATVIMLATGTGIAPFR.S + Oxidation (M)	+1
9	Chalcone syhthase	K.EAATKAIDEWGLPK.S	+1
10	Chlorophylll a, b, binding protein	R.ALEVIHGR.W<br>R.VDFKEPVWFK.A	+1
11	Chloroplast PSI Type III	K.WLAYGEVINGR.F<br>K.LGLIPAETALPWFK.T	+1
12	Plastidic aldolases	R.LASIGLENTEANR.Q<br>K.GLVPLAGSNDESWCQGLDGLASR.S<br>R.YAAISQDNGLVPIVEPEILLDGEHGIDR.T	+1
13	Ribosomal L12-1a	K.TEFDVVIDEVPSNAR.I	+1
14	P protein	R.FCDALISIR.Q<br>R.EYAAYPAPWLR.A	+1
16	NAD(P)-rossaman binding protein	K.AEQYLADSGIPYTIIR.A<br>R.KAEQYLADSGIPYTIIR.A	+1
17	Carbonic anhydrase	K.EAVNVSLGNLLTYPFVR.D<br>R.VCPSHVLDFQPGEAFVVR.N	+1
18	Dehydro ascorbate reductase	K.LYHLEIALGHYK.K	+1
19	Ascorbate peroxidase-2- like protein	K.YAADEDAFFADYAVSHMK.L	+1
20	Chlorophyll a, b,	R.FKESELIHCR.W	+1

	binding protein	K.YPGGAFDPLGYSK.D<br>K.KYPGGAFDPLGYSK.D	
21	ATP sythase β chain	R.IVQIIGPVHDVAFPPGK.M	+1
22	Ribulose-1,5-bisphosphate carboxylase activase	K.MCALFINDLDAGAGR.L<br>K.IVDTFPGQSIDFFGALR.A<br>R.VPIIVTGNDFSTLYAPLIR.D	+1
23	Glyceraldehyde-3-phosphate dehydrogenase	K.GLTAEDVNAAFR.K<br>K.KGLTAEDVNAAFR.K<br>R.KDSPLEVVVVNDSGGVK.N<br>K.VVAWYDNEWGYSQR.V	+1
24	Rieske-FeS protein	K.GDPTYLVVENDK.T	+1
25	SGRP glycine rich binding protein	R.GFGFVTFK.D<br>R.NITVNEAQSR.G<br>R.EGGGGGYGGGGGYGGR.R	+1
26	Maturase K	R.IHFYEKIK.H<br>K.VQFCNAAGHPISK.S<br>R.SQMLENSFIMDNAMKK.L + Oxidation (M)	+1
27	Carbonic anhydrase 3	K.EAVNVSLGNLLTYPFVR.D<br>R.VCPSHVLDFQPGEAFVVR.N	+1
28	Leafy like protein	-.HPFIVTEPGEVAR.G	+1
29	Chloroplast heat shock protein 70-1	K.AVVTVPAYFNDSQR.T<br>R.IINEPTAASLAYGFER.K<br>K.LSFSDIDEVILVGGSTR.I<br>K.SEVFSTAADGQTSVEINVLQGER.E	+1
30	ATP dependent Chloroplast protease putative	R.FNAQEALDYGLIDR.I	+1
31	S-adenosyl methionine synthase	R.SGAYIVR.Q<br>K.TAAYGHFGR.D<br>R.FVIGGPHGDAGLTGR.K<br>R.VHTVLISTQHDETVTNDEIAADLK.E<br>K.EHVIKPVIPAKYLDDNTIFHLNPSGR.F	+1
32	Predicted protein	R.SDKSVEALIMSK.E	+1
33	Hypothetical protein	K.FLLECCIDARYTMPNIPQDNGVAEMR.N + Oxidation (M)	+1
34	Fructose-bis-phospahte aldolase	K.EAAWGLAR.Y<br>R.SAAYYQQGAR.F<br>R.LASIGLENTEANR.Q	+1
35	MDR- ABC transporter like protein	K.TTLLEILAGK.V	+1
36	Plastidic aldolases	K.EAAWGLAR.Y<br>R.SAAYYQQGAR.F<br>R.LASIGLENTEANR.Q<br>K.GLVPLAGSNDESWCQGLDGLASR.S<br>R.YAAISQDNGLVPIVEPEILLDGEHGIDR.T	+1

37	Glyceraldehyde-3-phosphate dehydrogenase, putative	K.GLTAEDVNAAFR.K<br>K.KGLTAEDVNAAFR.K<br>R.KDSPLDVIVVNDSGGVK.N<br>K.VVAWYDNEWGYSQR.V	+1
38	Ferredoxin NADP+ oxidoreductase	K.EPYVGR.C<br>R.LVYTNDKGEEVK.G<br>R.LYSIASSALGDFGDSK.T<br>K.DPNATVIMLATGTGIAPFR.S<br>K.DPNATVIMLATGTGIAPFR.S + Oxidation (M)	+1
39	Malate dehydrogenase	R.DDLFNINAGIVK.S<br>K.ALEGADIVIIPAGVPR.K<br>K.LNPLVSSLSLYDIAGTPGVAADVSHINTR.S	+1
40	Carbonic anhydrase	K.YAGVGAAVEYAVLHLK.V<br>R.VCPSHVLDFQPGEAFVVR.N	+1
41	Ribose-5-phosphate-isomerase	R.IDLAIDGADEVDPDLNLVK.G	+1
42	Triosephosphate isomerase	K.TFDVCFK.Q<br>K.FFVGGNWK.C<br>K.VASPEQAQEVHVAVR.D	+1
43	Unknown protein	R.LMEFGRSR.V + Oxidation (M)	+1
44	Triosephosphate isomerase	K.FFVGGNWK.C<br>K.VASPEQAQEVHVAVR.D	+1

45	Hypothetical protein	R.EHGLTVSR.A<br>K.VSVPAAAVAR.A<br>K.KVSVPAAAVAR.A	+1
46	Unknown protein	R.NAETFCVGIMGR.S	+1

Supplementary Table S6: Results of BLAST P analysis

Spot no	Accession no (protein identified in Table 4)	Homologue			
		**Accession no** [a]	**Protein name** [b]	**Species**	**% similarity** [c]
1	gi|68052991	O20250.1	Transketolase	*Spinacia oleracea*	64
2	gi|115813	XP_002273201.1	Chlorophylll a, b, binding protein	*Vitis vinifera*	68
3	gi|89475526	ABD73295.1	Photosynthetic e-transfer like protein	*Panax ginseng*	64
4	gi|131390	P16059.1	Oxygen evolving complex protein 2	*Pisum sativum*	64
5	gi|21283	NP_001043134.1	Oxygen evolving complex protein 2	*Oryza sativa japonica*	100
6	gi|269856436	ACZ51445.1	Glyceraldehyde-3-phosphate dehydrogenase	*Mikania micrantha*	54
8	gi|61969078	NP_001105568.1	Ferredoxin NADP+ oxidoreductase	*Zea mays*	48
9	gi|1403057	CAA62686.1	Chalcone synthase	*Leibnitzia anandria*	100
10	gi|20671	XP_002525758	Chlorophylll a, b, binding protein	*Ricinus communis*	60
11	gi|159138839	ABW89422.1	Chloroplast PSI Type III Chlorophylll a, b, binding protein	*Helianthus annuas*	55
12	gi|1781348	ADD52195.1	Plastidic aldolase	*Catharanthus roseus*	50
13	gi|20020	CAA44226.1	Ribosomal protein L12	*Nicotiana tabacum*	100
14	gi|438003	P49361.1	Glycine cleavage P protein	*Flaveria pringlei*	57
16	gi|18404496	NP_565868.1	NAD(P)-rossaman binding protein	*Arabidopsis thaliana*	100
17	gi|1089983	ACZ74707.1	Carbonic anhydrase	*Phaseolus vulgaris*	54
18	gi|66732627	AFF18803.1	Dehydroascorbate reductase	*Dimocarpus longan*	100

19	gi|290796648	ADD64889.1	Ascorbate peroxidase -2-like protein	*Tragopogun dubius*	100
20	Unknown protein	XP_002533251.1	Chl a,b binding protein putative	*Ricinus communis*	51
21	gi|7688419	CAB90006.1	ATP synthase B chain	*Buddleja auriculata*	100
22	gi|358249078	CAO02533.1	Rubisco activase	*Vigna Unguiculata*	35
23	gi|255641007	P09044.1	Glyceraldehyde-3-phosphate dehydrogenase	*Nicotiana tabacum*	100
24	gi|9843639	NP_192237.1	Rieske-FeS protein	*Arabidopsis thaliana*	100
25	gi|544426	AAG23220.1	SGRP glycine rich binding protein	*Sorghum bicolor*	41
26	gi|290583766	AAL36004.1	Maturase K	*Oemlaria cerasiformiss*	49
27	gi|1089983	ACZ74707.1	Carbonic anhydrase	*Phaseolus vulgaris*	51
28	gi|14573451	AAS79843.1	FLO/LFY like protein	*Berlinia confusa*	100
29	gi|166919370	ABZ04080	Chloroplast heat shock protein	*Ipomoea nil*	60
30	gi|255537123	XP_002509628.1	ATP dependent chloroplast protease	*Ricinus communis*	100
31	gi|15228048	Q94FA5.1	S-adenosiyl methionine synthase	*Brassica juncea*	33
32	gi|224130876	XP_002328398.1	Unknown protein	*Populus trichorpa*	100
33	gi|147845220	CAN81611.1	Unknown protein	*Vitis vinifera*	100
34	gi|22633	XP_003631873	Fructose-bis-phosphate aldolase	*Vitis vinifera*	70
35	gi|224090097	NP_175734.1	ABC transporter G familyprotein	*Arabidopsis thaliana*	100
36	gi|1781348	BAA77603.1	Plastidic aldolase	*Nicotiniia planiculata*	46

37	gi|255539282	XP_002510706.1	Glyceraldehyde-3-phosphate dehydrogenase	*Ricinus communis*	37
38	gi|61969078	O04977.1	Ferredoxin NADP+ oxidoreductase	*Nicotiana tabacum*	51
39	gi|21388544	AAF69802.1	Malate dehydrogenase	*Vitis vinifera*	51
40	gi|339958979	AEK25173.1	Carbonic anhydrase	*Dimocarpus longan*	55
41	gi|18654317	BAC83440.1	Ribose-5-phosphate isomerase	*Oryza sativa japonica*	100
42	gi|1351282	P48497.1	Triose phosphate isomerase	*Stellaria longipes*	48
43	gi|302844664	XP_002953872.1	Hypothetical protein	*Volvox cartei*	100
44	gi|1351282	P48497.1	Triose phosphate isomerase	*Stellaria longipes*	100
45	gi|242061954	XP_002452266.1	Hypothetical protein	*Sorghum bicolor*	100
46	gi|296081316	CBI17698.3	Unknown protein product	*Vitis vinifera*	100

BLASTP (http://www.ncbi.nlm.nih.gov/BLAST/) was used to search for homologues of the unknown proteins and to validate the results
The peptide with maximum score was also validated by BLAST P analysis (Table 3)
[a] Acc. no., The accession number of the unknown proteins in Table 3.
[b] Acc. no., The accession number of the homologues.
[C]% similarity.

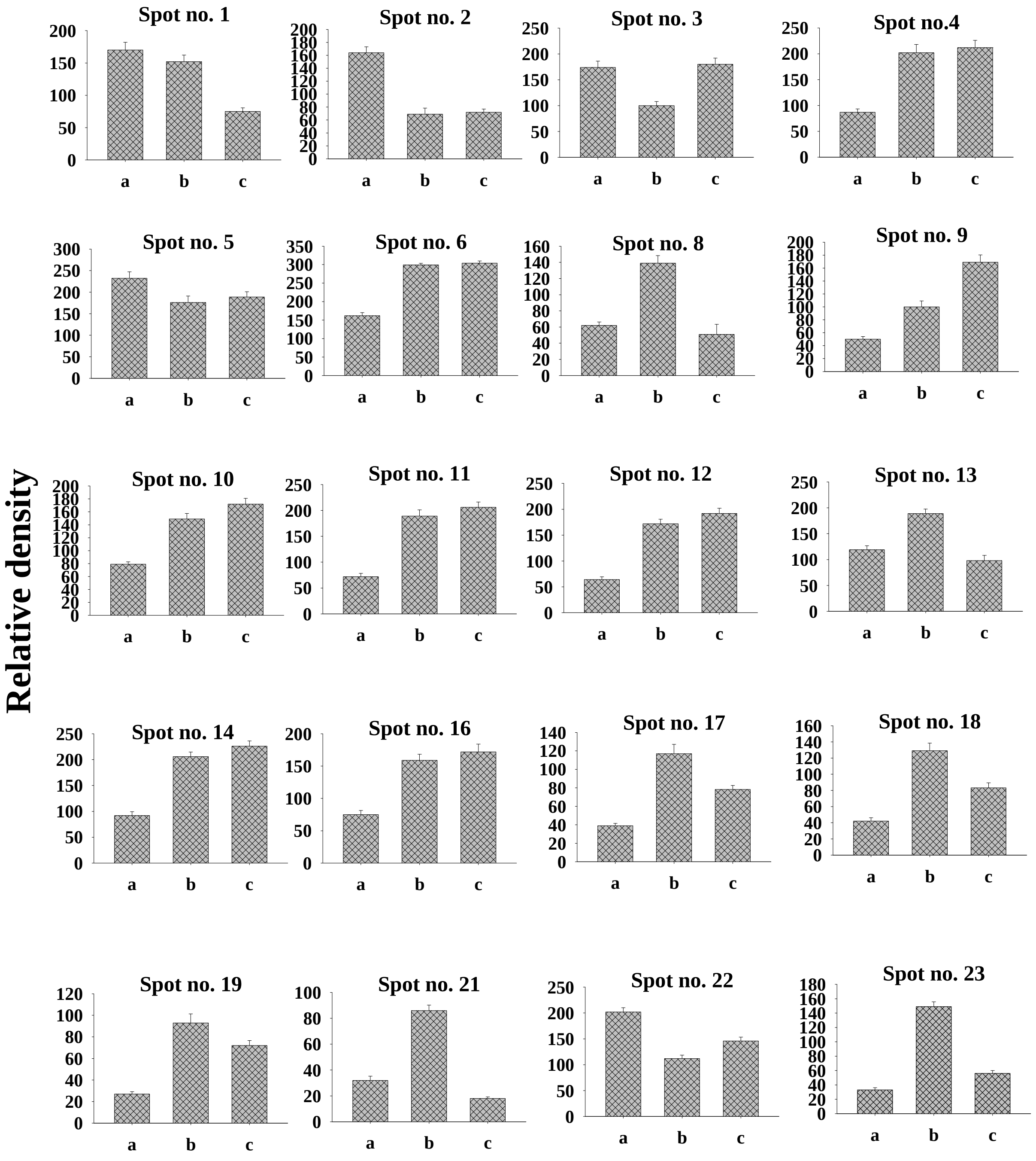
Relative density
Spot no. 1
Spot no. 2
Spot no. 3
Spot no.4
Spot no. 5
Spot no. 6
Spot no. 8
Spot no. 9
Spot no. 10
Spot no. 11
Spot no. 12
Spot no. 13
Spot no. 14
Spot no. 16
Spot no. 17
Spot no. 18
Spot no. 19
Spot no. 21
Spot no. 22
Spot no. 23
a
b
c

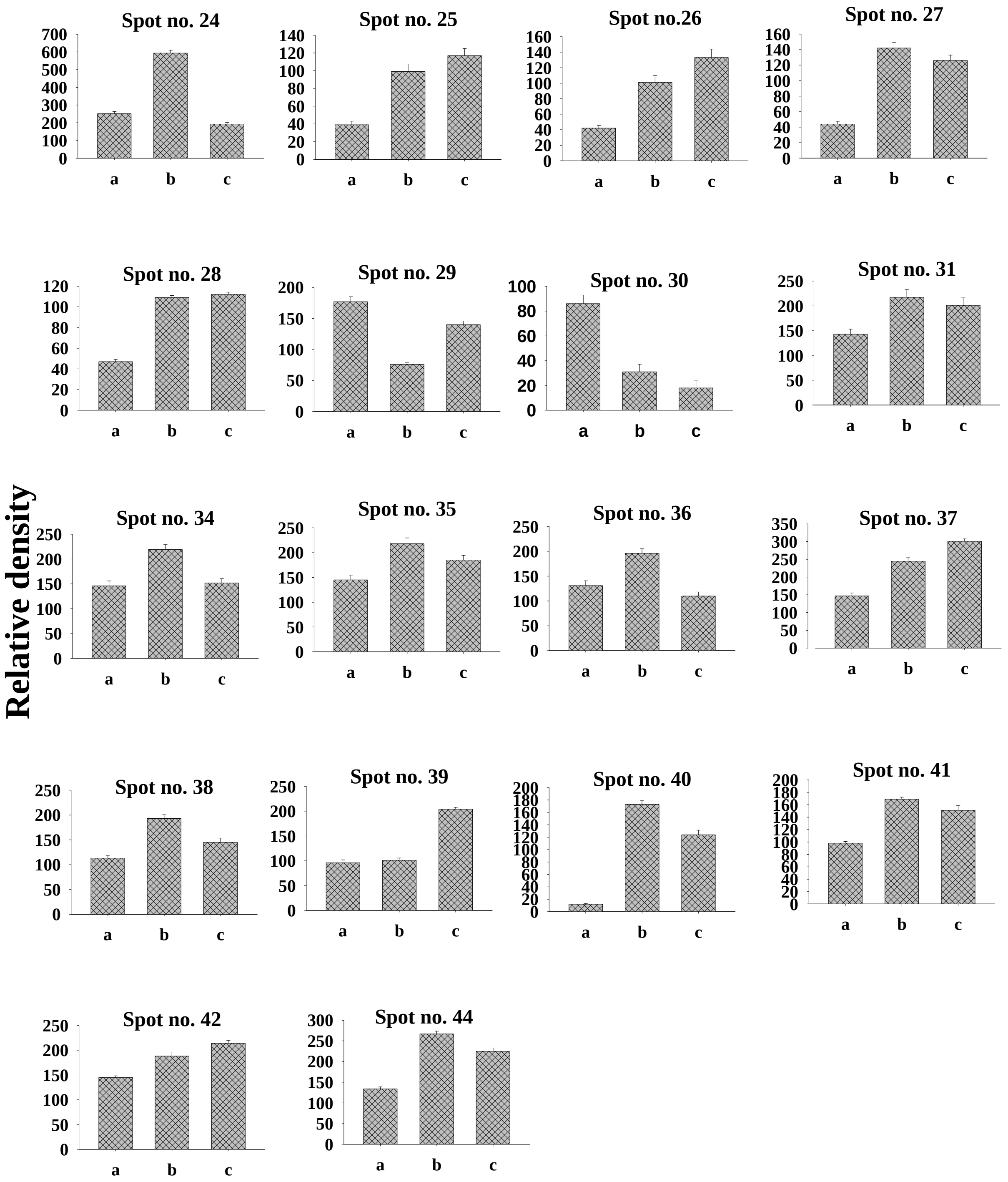

Supplementary Fig. S1- Relative intensity of identified protein spots; control (a), 100 µM As (b); and 150 µM As respectively. Values are means±SD of spot volumes of gels from three independent experiments. Serial numbers correspond to spots numbers on the 2-DE gel.

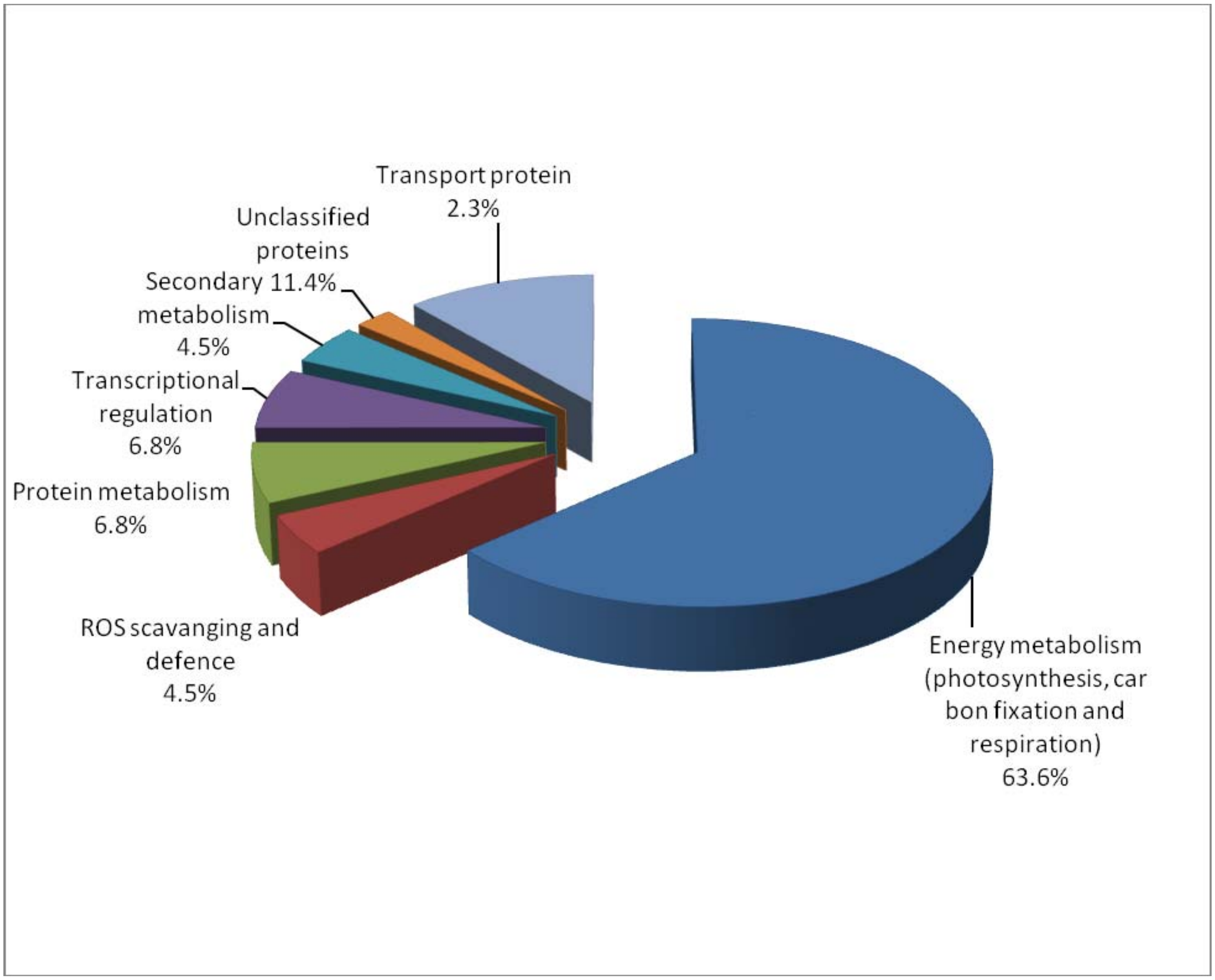

Supplementary Fig. S2 Functional classification and distribution of 46 identified proteins from 2-DE gel of As-treated *Artemisia annua.*

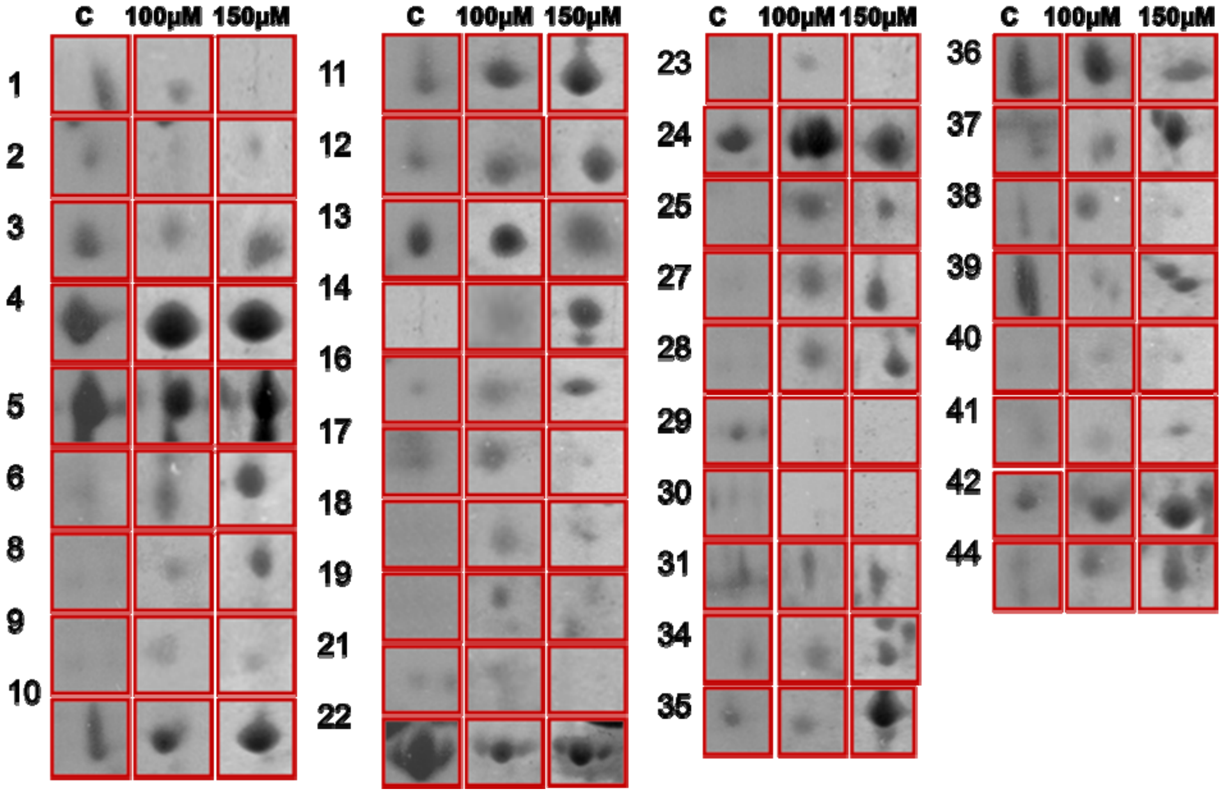

Supplementary Fig. S3-Expression pattern of selected proteins of C, control and; 100, 150µM As treatment given to *Artemisia annua*. Numbers represent the spots no. on 2DE gel.
